# Can Temperature and UV Exposure Alter the Antimicrobial Properties of the Essential Oils? An Investigation of *Origanum vulgare*, *Cymbopogon citratus*, *Syzygium aromaticum*, and *Lippia sidoides*

**DOI:** 10.1021/acsomega.6c06182

**Published:** 2026-09-16

**Authors:** Ramon Peres Brexó, Conny Wara Teran Fukuyama, Giulia Suguimoto Martins, Maria Eduarda da Mata Martins, Fernanda Ramalho Procopio, Stanislau Bogusz Junior, Marcos David Ferreira

**Affiliations:** † Brazilian Agricultural Research Corporation, 67838 Embrapa Instrumentation, São Carlos 13561-206, Brazil; ‡ Center for Biological and Health Sciences, 67828 Federal University of São Carlos (UFSCar), São Carlos 13565-905, São Paulo, Brazil; § Postgraduate Program in Food Science and Engineering, 28108 IBILCE, São Paulo State University “Julio de Mesquita Filho”, UNESP, São José do Rio Preto 15054-000, São Paulo, Brazil; ∥ University of São Paulo (USP), São Carlos Institute of Chemistry (IQSC), São Carlos 13566-590, São Paulo, Brazil

## Abstract

Essential oils (EOs) are natural compounds widely applied in agriculture, post-harvest preservation, food processing, and human health due to their strong antimicrobial potential. However, the effects of postextraction physical treatments on their chemical and biological properties remain underexplored. This study investigated the effects of 48 h of exposure to heat at 70 °C and UV radiation on EOs from *oregano* (OV), clove (SA), lemongrass (CC), and the Brazilian native pepper-rosmarin (LS). Chemical profiles were determined by GC–MS, while antimicrobial activities were assessed against *Escherichia coli*, *Staphylococcus aureus*, *Colletotrichum* sp., and *Fusarium solani*. Both treatments altered EOs chemical compositions, particularly in OV and LS. Interestingly, some antimicrobial activity increased after the treatments, with more consistent improvements observed against fungal than bacterial strains. In addition, micrographs revealed structural damage to the fungal hyphae caused by the treated LS EO, suggesting different effects on hyphal morphology. These findings demonstrate that controlled postextraction physical treatments can modulate the chemical composition and antimicrobial activity of EOs in a specific and controlled manner. This practical approach provides a simple and potentially effective strategy for tailoring EOs bioactivity and supports further investigation of physical processing as a tool for enhancing naturally derived antimicrobial systems.

## Graphical abstract

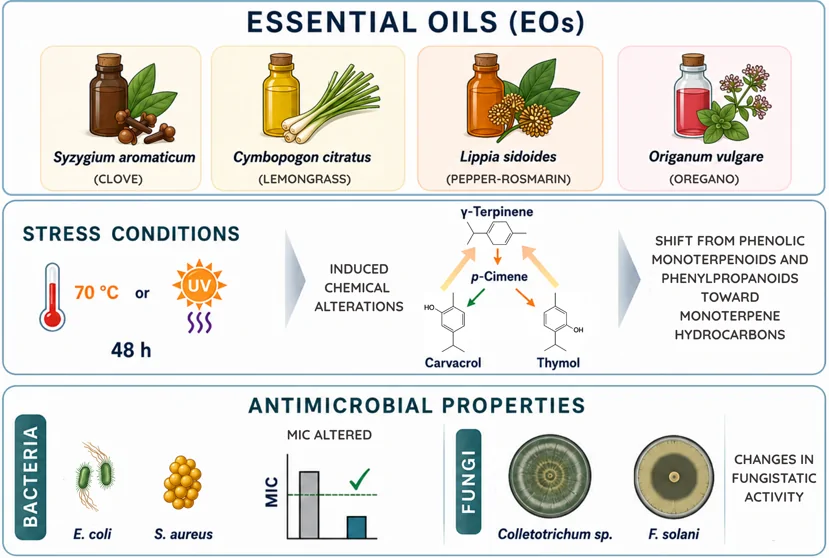

## 1 Introduction

Essential oils have been extensively studied in recent years due to their diverse applications across multiple sectors, including human health, agriculture, postharvest preservation, and food processing. Emerging applications include their incorporation into biodegradable packaging films, functional foods designed to enhance well-being, edible coatings for food products, and their use as active ingredients in nano- and microencapsulation systems or as carriers in nanoemulsions.^1–4^ The chemical composition and bioactivity of essential oils result from a complex interplay between the intrinsic and extrinsic factors. Among the intrinsic factors, the genetic profile of the plant species, gene expression, developmental stage, and enzymatic regulation of specific metabolic pathways stands out. For example, terpene synthases play a central role in the biosynthesis of mono- and diterpenes, whereas phenylalanine ammonia lyase is crucial in the production of phenylpropanoids. These secondary metabolites, stored in specialized structures such as glandular trichomes, secretory cavities, and resin ducts, perform essential biological roles, ranging from pollinator attraction to defense against environmental stressors.^5–7^

Extrinsic factors also exert a significant influence on both the quality and the quantity of EOs. Morphoclimatic conditions such as circadian rhythm and seasonality strongly affect the EO chemical profile.^8,9^ Abiotic conditions, including temperature, drought, solar radiation, and even the presence of heavy metals, modulate the biosynthesis of volatile metabolites.^8,10,11^ Furthermore, postharvest processing, particularly the drying method of plant material, is decisive. For example, hot-air drying may cause degradation or volatilization of sensitive compounds, whereas lyophilization better preserves the integrity of the chemical profile.^12–14^ Although genetic, environmental, and technological factors affecting the chemical profiles of EOs have been widely studied, the influence of postextraction physical treatments on their composition and bioactivity remains largely overlooked. For controlled heating exposure, only limited and emerging evidence is available in the literature,^15–19^ whereas, to the best of our knowledge, no studies have yet investigated the effects of direct UV irradiation on the chemical composition and bioactivity of EOs.

In this context, treatment-induced chemical modifications in EOs may modulate their antimicrobial activities. This phenomenon represents a promising strategy for controlling phytopathogenic fungi, including *Colletotrichum* spp. and *Fusarium* spp., which contribute to postharvest losses.^20–23^ It may also enhance the control of human pathogenic bacteria, such as *Escherichia coli* and *Staphylococcus aureus*.^24–26^ *F. solani* is a cosmopolitan soil-borne fungus that can act as both a saprophyte and a destructive plant pathogen. Beyond its association with root and vascular diseases in diverse hosts, it is also a major agent of postharvest decay in tropical fruits such as papaya, mango, avocado, and pineapple.^22^ Infected fruits often exhibit brown to necrotic lesions that progress to wilting and tissue softening, which drastically reduce shelf life and marketability.^22,27^ Management of *F. solani* is particularly challenging, as conventional fungicides are not always effective and may cause undesirable ecological impacts.^28^ Similarly, species of *Colletotrichum* are among the most devastating postharvest pathogens worldwide, infecting a broad range of fruit crops such as mango, apple, and strawberry.^29^ Characterized by their ability to induce anthracnose symptoms, these fungi cause tissue necrosis and fruit rot, resulting in substantial economic losses during postharvest storage and handling.^27,29,30^ Given their relevance as resistant plant pathogens, these fungi were selected as target organisms in this study to evaluate the antifungal efficacy of the EOs. Moreover, the well-documented antimicrobial properties of EOs against Gram-positive bacteria such as *S. aureus* and Gram-negative bacteria such as *E. coli* further emphasize their potential as versatile bioactive agents.^25,26,31,32^ Therefore, postextraction treatments of EOs may represent a valuable strategy for enhancing their applications in medicine, food preservation, and agriculture.

Based on the above considerations, the present study evaluated the impact of physical treatments (heating at 70 °C and UV exposure for 48 h) on the EOs from oregano (*Origanum vulgare*), clove (*Syzygium aromaticum*), and lemongrass (*Cymbopogon citratus*), which are non-native to Brazil, and pepper-rosmarin (*Lippia sidoides*), a Brazilian native species that remains underexplored as an antimicrobial agent. The assessment encompassed chemical composition, determination of minimum inhibitory concentration (MIC) against Gram-positive and Gram-negative bacteria, as well as effects on mycelial growth (supported by SEM imaging) and inhibition of conidial germination of the phytopathogenic fungi *Colletotrichum* sp. and *Fusarium solani.*

## 2 Results and Discussion

### 2.1 Visual Alteration in Thermal and Photo-UV EOs Exposed

Regarding the original color of the EOs, *C. citratus* (Figure 1B) exhibited the most distinct hue, showing a more intense yellow tone. In contrast, the other essential oils appeared more transparent with subtle shades ranging from yellowish to greenish or light brown.

**Figure 1:**
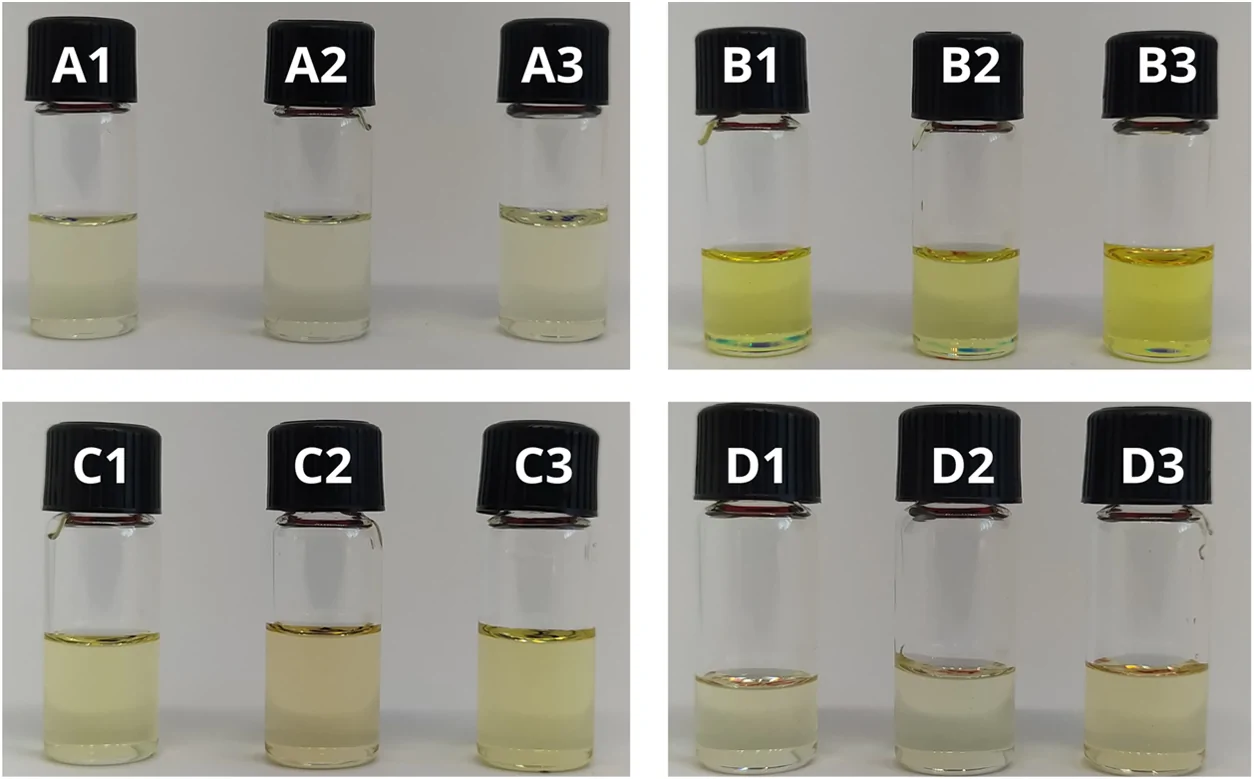
Visual appearance of essential oil samples after different treatments. A—*Origanum vulgare*, B—*Cymbopogon citratus*, C—*Lippia sidoides*, D—*Syzygium aromaticum*. For each essential oil: 1—intact (untreated), 2—photo-UV treated, and 3—thermally treated.

The color changes in samples C2 (*L. sidoides*) and D3 (*S. aromaticum*) were the most pronounced compared to their untreated controls, as shown in Figure 1. Additionally, according to the colorimetric data presented in Table 1, these differences were corroborated by higher total color difference (ΔE*) values, particularly for SA-T (ΔE* = 4.97), LS-P (ΔE* = 4.15), and LS-T (ΔE* = 3.96).

**Table 1:** Colorimetric Data of EOs after 48 h of Thermal (-T) and Photo-UV (-P) Stress Exposure^a^

	OV	OV-P	OV-T
L*	37.84 ± 0.28 ^A^	38.14 ± 0.12 ^A^	37.79 ± 0.54 ^A^
a*	–1.74 ± 0.20 ^A^	–1.68 ± 0.07 ^A^	–1.82 ± 0.15 ^A^
b*	10.27 ± 0.21 ^A^	8.31 ± 0.09 ^C^	9.09 ± 0.28 ^B^
Δ*E*		**1.99**	**1.19**

	CC	CC-P	CC-T
L*	36.44 ± 0.38 ^B^	36.64 ± 0.25 ^B^	37.31 ± 0.15 ^A^
a*	–4.74 ± 0.17 ^B^	–4.20 ± 0.04 ^A^	–3.95 ± 0.09 ^A^
b*	21.20 ± 0.59 ^A^	18.35 ± 0.26 ^B^	20.33 ± 0.22 ^A^
Δ*E*		**2.91**	**1.46**

	LS	LS-P	LS-T
L*	37.59 ± 0.73 ^A^	35.91 ± 0.28 ^B^	34.95 ± 0.51 ^B^
a*	–1.74 ± 0.24 ^B^	1.10 ± 0.03 ^A^	–1.59 ± 0.11 ^B^
b*	14.09 ± 0.62 ^B^	16.61 ± 0.25 ^A^	17.04 ± 0.50 ^A^
Δ*E*		**4.15**	**3.96**

	SA	SA-P	SA-T
L*	39.12 ± 0,26 ^A^	37.95 ± 0.44 ^B^	36.64 ± 0.55 ^C^
a*	–0.87 ± 0.07 ^B^	–0.82 ± 0.11 ^B^	–0.09 ± 0.09 ^A^
b*	6.49 ± 0.11 ^B^	6.90 ± 0.22 ^B^	10.72 ± 0.36 ^A^
Δ*E*		**1.25**	**4.97**

a OV: *Origanum vulgare* intact (untreated); OV-P: *O*. *vulgare* photo-UV treated; OV-T: *O*. *vulgare* thermally treated. CC: *Cymbopogon citratus* intact (untreated); CC-P: *C*. *citratus* photo-UV treated; CC-T: *C*. *citratus* thermally treated; LS: *Lippia sidoides* intact (untreated); LS-P: *L*. *sidoides* photo-UV treated; LS-T: *L*. *sidoides* thermally treated; SA: *Syzygium aromaticum* intact (untreated); SA-P: *S*. *aromaticum* photo-UV treated; SA-T: *S. aromaticum* thermally treated. Superscript capital letters indicate statistically significant differences according to ANOVA and Tukey’s test (*p* ≤ 0.05) when comparing intact, photo-UV-treated, and thermally treated EOs.

According to Gonnet,^33^ the human eye can perceive color differences when ΔE* exceeds 1. Therefore, all visual changes caused by exposure to elevated temperature and UV radiation in the present study are noticeable. A previous study with eucalyptus oil, lavender oil, pine oil, turpentine oil, may chang oil, rosemary oil, and thyme oil stored at 38 °C under cool white light in the presence of atmospheric oxygen for periods exceeding 4 weeks also reported ΔE* values greater than 1 for all EOs tested, indicating that color shifts also during storage were distinguishable to the human eye.^34^ Specifically, rosemary and lavender oils exhibited a positive correlation between ΔE* and storage time, whereas may chang and thyme oils initially developed a pronounced yellow hue that later shifted to a livid tone upon prolonged exposure to heat and light. In agreement with these findings, our results show that the magnitude of color change was more prominent for specific samples, particularly *L. sidoides* and *S. aromaticum*. This suggests that the response to physical stress, such as elevated temperature and UV exposure, does not affect the color of EOs uniformly. Furthermore, the higher ΔE* values observed for certain treatments may indicate physicochemical changes in the EOs during exposure, potentially reflecting alterations in their chemical composition and bioactivity. This interpretation is supported by previous observations for clove bud (*S. aromaticum*) EO, where prolonged storage and oxidation resulted in darkness. These observation were associated with changes in the relative contents of caryophyllene, eugenyl acetate, and other compounds.^35^ Although color variation alone cannot establish the nature or extent of these changes, it may serve as a simple, visually detectable, and readily measurable initial parameter for screening essential oil stability and identifying samples that warrant further chemical and functional investigation. To further assess whether color variation is directly associated with changes in other characteristics of the EOs following heat and UV treatments, specific compositional changes and antimicrobial activity were investigated and are discussed in the following sections.

### 2.2 EO Chemical Alterations

The chemical composition of all analyzed EOs is presented in Table 2, and the corresponding chromatograms are shown in the Supporting Information as Figures S1, S2, S3, and S4. According to the data, the *O. vulgare* essential oil (OV) initially contained a high proportion of carvacrol (79.93%), and after exposures, the major compounds were *p*-cymene (OV-T: 39.43%; OV-P: 37.29%), carvacrol (OV-T: 20.84%; OV-P: 20.35%), and γ-terpinene (OV-T: 18.15%; OV-P: 19.68%). In *L. sidoides* essential oil (LS), the most abundant compounds in the untreated essential oil were thymol (61.90%), caryophyllene (10.56%), and *p*-cymene (9.27%). However, in the treated LS, *p*-cymene (LS-T: 38.93%; LS-P: 40.81%), γ-terpinene (LS-T: 13.25%; LS-P: 11.94%), and thymol (LS-T: 11.76%; LS-P: 11.84%) became predominant. In *C. citratus* essential oil (CC), β-citral (CC: 42.46%; CC-T: 45.71%; CC-P: 44.75%), α-citral (CC: 33.27%; CC-T: 34.22%; CC-P: 34.39%), and myrcene (CC: 10.03%; CC-T: 3.87%; CC-P: 3.95%) were identified as the main components, showing high significant changes on the last volatile component. Lastly, the essential oil from *S. aromaticum* (SA) was dominated by eugenol (SA: 84.50%; SA-T: 82.54%; SA-P: 82.46%) and caryophyllene (SA: 11.68%; SA-T: 12.03%; SA-P: 11.62%), which remained relatively stable after both treatments.

**Table 2:**
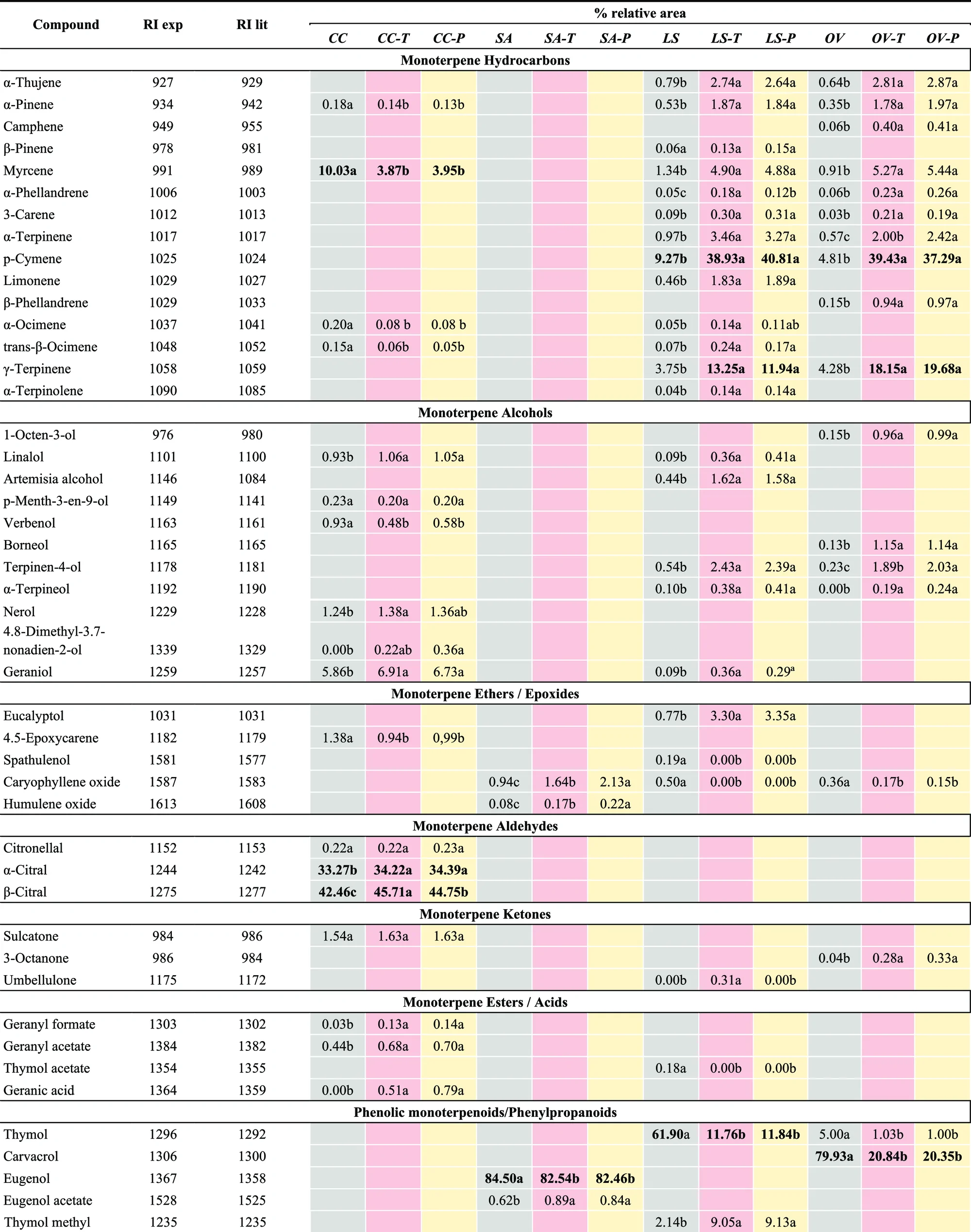

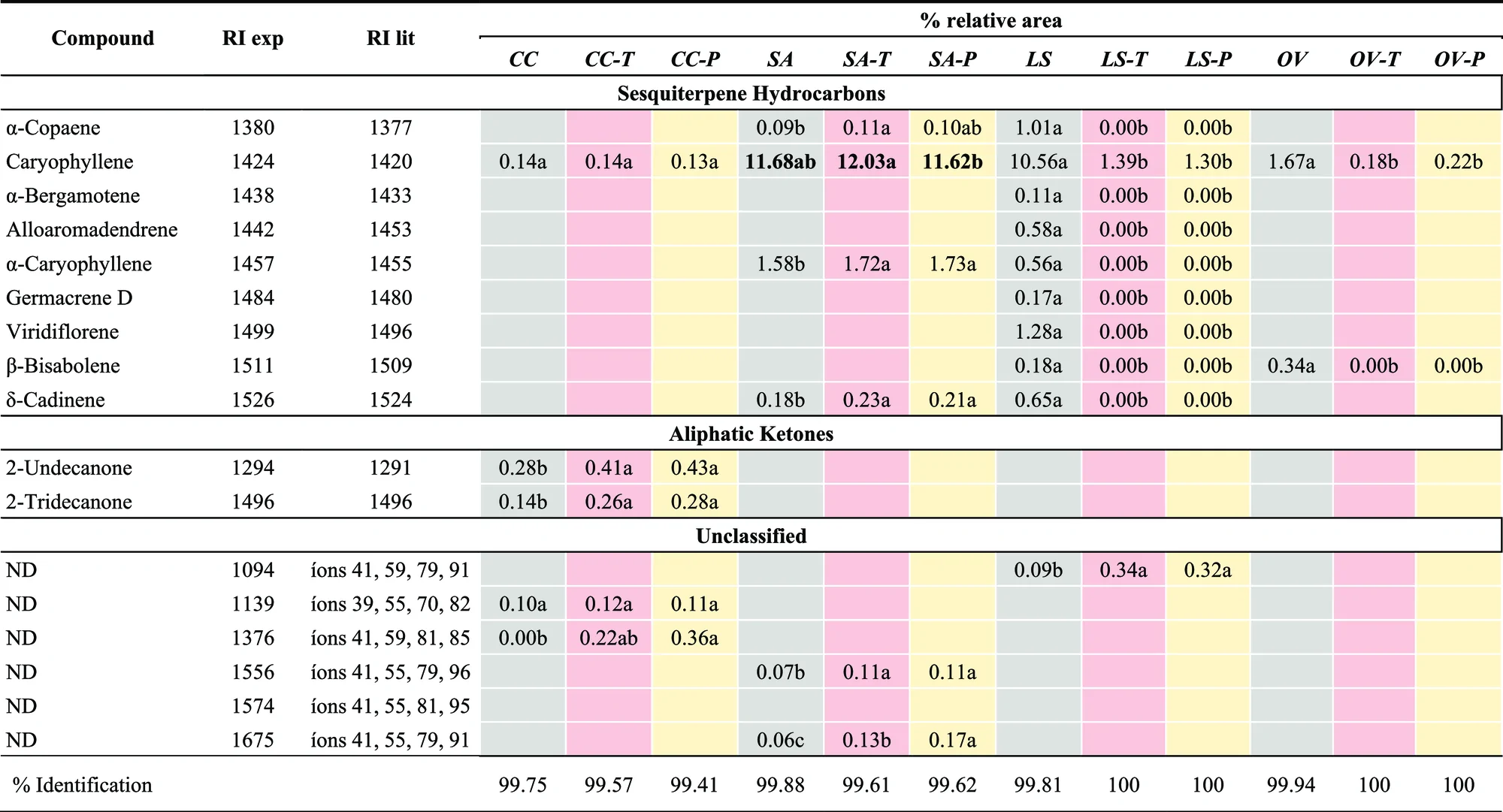
Identification and Semiquantification of EO Constituents by GC-MS and GC-FID^a^

a OV: *Origanum vulgare* intact (untreated); OV-P: *O. vulgare* photo-UV treated; OV-T: *O. vulgare* thermally treated. CC: *Cymbopogon citratus* intact (untreated); CC-P: *C. citratus* photo-UV treated; CC-T: *C. citratus* thermally treated; LS: *Lippia sidoides* intact (untreated); LS-P: *L. sidoides* photo-UV treated; LS-T: *L. sidoides* thermally treated; SA: *Syzygium aromaticum* intact (untreated); SA-P: *S. aromaticum* photo-UV treated; SA-T: *S. aromaticum* thermally treated. Gray columns indicate intact (untreated) essential oils (EOs), red columns represent thermally treated EOs, and yellow columns represent photo-UV-treated EOs. ND: not detected. Compounds listed in order of elution from DB-5MS capillary column; RI exp: retention index experimental; RI lit: retention index from the literature (National Institute of Standards and Technology (https://webbook.nist.gov/chemistry) and/or Pherobase (https://pherobase.com/)). Means with different letters on the same line differ significantly (*p* ≤ 0.05) by ANOVA and Tukey’s test. The mean relative area percentages of the major compounds of each essential oil are highlighted in bold.

Notably, in all cases where the EOs exhibited the compositional changes induced by either thermal treatment or UV exposure, the shifts among chemical classes followed similar patterns, as shown in Figure 2.

**Figure 2:**
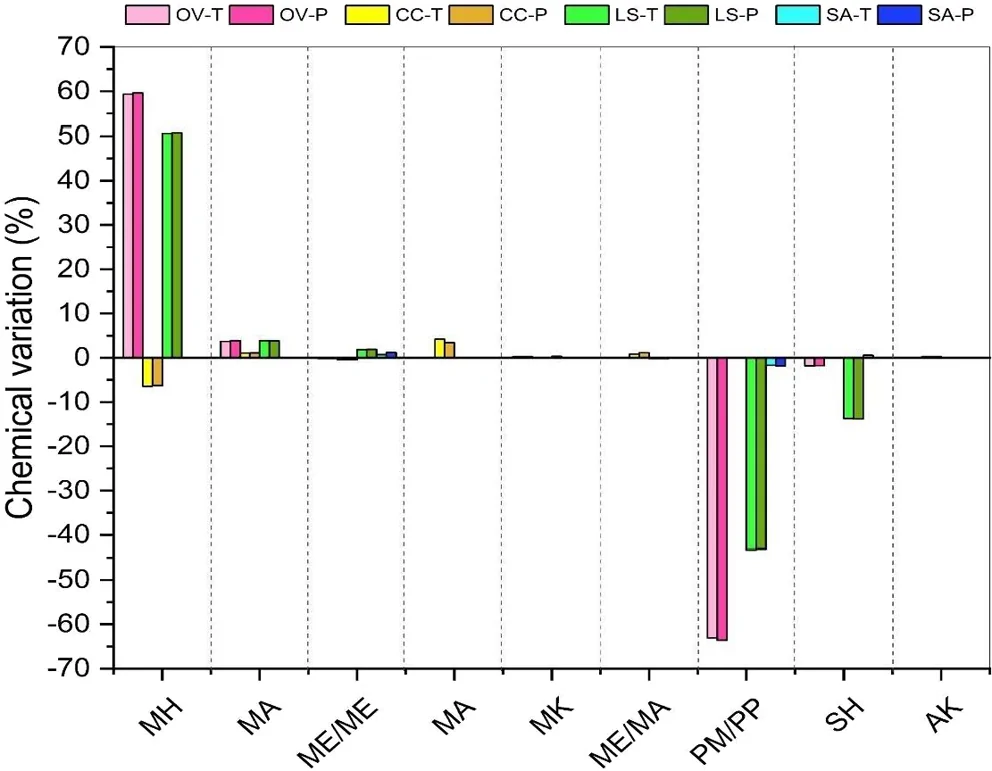
Percentage variation in the concentration of chemical classes in the composition of the EOs. OV: *Origanum vulgare* intact (untreated); OV-P: *O. vulgare* photo-UV treated; OV-T: *O. vulgare* thermally treated. CC: *Cymbopogon citratus* intact (untreated); CC-P: *C. citratus* photo-UV treated; CC-T: *C. citratus* thermally treated; LS: *Lippia sidoides* intact (untreated); LS-P: *L. sidoides* photo-UV treated; LS-T: *L. sidoides* thermally treated; SA: *Syzygium aromaticum* intact (untreated); SA-P: *S. aromaticum* photo-UV treated; SA-T: *S. aromaticum* thermally treated. The chemical classes evaluated are as follows: monoterpene hydrocarbons (MH), monoterpene alcohols (MA), monoterpene ethers/epoxides (ME/ME), monoterpene aldehydes (MA), monoterpene ketones (MK), monoterpene esters/acids (ME/MA), phenolic monoterpenoids/phenylpropanoids (PM/PP), sesquiterpene hydrocarbons (SH), and aliphatic ketones (AK). Values represent the relative concentration variation (%) compared to untreated EOs.

The compounds were grouped into the following classes of organic compounds: monoterpene hydrocarbons (MH), monoterpene alcohols (MA), monoterpene ethers/epoxides (ME), monoterpene aldehydes (MA), monoterpene ketones (MK), monoterpene esters/acids (MEs), phenolic monoterpenoids/phenylpropanoids (PP), sesquiterpene hydrocarbons (SH), and aliphatic ketones (AK). Based on the relative percentage area of the untreated EOs, the decreasing order of constituent variation after thermal and photo-UV treatments was: *Origanum vulgare* (OV) > *Lippia sidoides* (LS) > *Cymbopogon citratus* (CC) > *Syzygium aromaticum* (SA). This result confirms that the visual alterations observed in EOs subjected to thermal and photo-UV exposure do not directly reflect their chemical changes. This lack of direct correlation becomes even more evident when examining the data for thermally treated *S. aromaticum* essential oil, which exhibited a ΔE* of 4.97, yet showed only a 2.95% variation in composition compared to the untreated oil.

Among the EOs, *O.* *vulgare* and *L. sidoides* exhibited the most significant compositional changes, with a marked shift from phenylpropanoids to monoterpene hydrocarbons following exposure. *L. sidoides* also showed a reduction in the level of sesquiterpene hydrocarbons. In contrast, *C. citratus* exhibited a different trend, characterized by decreased monoterpene hydrocarbons and increased monoterpene aldehydes. For *S. aromaticum*, only minor changes were observed, suggesting that the constituents of this oil are more resistant to the physical exposure conditions evaluated in this study.

Olmedo et al.^17^ reported changes in the composition of Argentine oregano essential oil stored at 60 °C for 28 days. Among these alterations, terpinen-4-ol decreased from 29.5 to 26.0%, linalool from 13.1 to 5.7%, while carvacrol increased from 20.4 to 26.4%, and ortho-cymene rose markedly from 1.94 to 13.13%. In this case, the most pronounced change also occurred for a monoterpene hydrocarbon (ortho-cymene). In addition, that study also compared compositional changes in rosemary, and laurel EOs treated at 60 °C for 28 days. All terpenes exhibited degradation under the tested conditions, with camphor, citronellal, and carvacrol degrading more slowly than α-pinene and limonene. The authors suggested that differences in boiling points could account for these distinct thermal degradation rates. However, no consistent relationship was observed between the thermal stability of individual molecules across different EOs. For instance, α-pinene levels decreased in rosemary essential oil but increased in oregano and laurel EOs. Such discrepancies may be linked to interactions among the various constituents in oregano, rosemary, and laurel oils. In addition, Corradi et al.,^16^ similarly demonstrated that prolonged thermal exposure can alter the chemical composition of lemongrass (*Cymbopogon flexuosus*) essential oil and consequently affect its functional and sensory properties. After storage at 60 °C for 60 days, β-citral decreased from 41.53 to 32.01%, whereas α-citral increased from 32.85 to 40.83%, resulting in a decrease in the β-citral/α-citral ratio from 1.26 to 0.78. These compositional changes were accompanied by a reduction in antioxidant performance and aroma intensity, with 30 days of thermal exposure suggested as a practical limit for maintaining acceptable sensory quality. These findings further support that prolonged heating can selectively modify EO composition, with direct consequences for their functional and sensory properties.

In the present study, molecular interactions between major and minor components may also help explain the stability observed in the EO. The number of volatile constituents present in the intact EOs is proportional to the chemical differences detected after exposure to physical stressors, such as UV light and heat. This suggests that the richer an intact essential oil is in a few dominant components, the greater its stability is against such physical agents. In this case, *S. aromaticum* essential oil initially contained 10 volatile constituents, with the major compounds accounting for 96.18% of the total composition (Table 2). In contrast, *O.* *vulgare* essential oil had 21 volatile constituents, with the major compound representing 79.93%, and *L. sidoides* essential oil contained 35 volatile constituents, with the major compounds comprising 81.26% of the total. In other words, the findings of this study indicate that the greater the diversity of compounds, as observed in *O.* *vulgare* and *L. sidoides* EOs, the more extensive the chemical changes that may occur. According to established principles of chemical kinetics (collision theory and the law of mass action), the reaction rate typically increases with the number and hence the concentration of reactants, since more molecules raise the likelihood of collisions.^36^ Furthermore, according to the Arrhenius equation, increasing the temperature or energy input (e.g., thermal or UV exposure) elevates the proportion of collisions that can overcome the activation energy barrier, thus, enhancing reaction rates.

The biosynthesis of thymol and carvacrol in *O. vulgare* and *L. sidoides* originates from the universal monoterpene precursor geranyl pyrophosphate (GPP). GPP undergoes cyclization to form the α-terpinyl cation, an unstable carbocationic intermediate that can be directed toward specific monoterpene products by terpene synthase (TPS) enzymes. Among these, γ-terpinene synthase (Tps2 or Tctps2) catalyzes the formation of γ-terpinene and *p*-cymene, both recognized as key precursors for phenolic monoterpenes such as thymol and carvacrol.^37–40^ In *O. vulgare*, the expression of the γ-terpinene synthase gene OvTPS2 has been correlated with higher thymol and carvacrol accumulation in leaves, highlighting the central role of this enzyme in the phenolic monoterpene pathway. Although *p*-cymene has been proposed as an intermediate in the conversion of γ-terpinene to thymol and carvacrol, the precise enzymatic steps responsible for aromatic ring formation and hydroxylation remain incompletely elucidated.^41^

In plant cells, the biosynthetic pathway is energetically driven by enzyme-catalyzed reactions that lower the activation energy of key transformations. Under physiological conditions, this results in a net conversion from monoterpene hydrocarbons (MH) such as γ-terpinene and *p*-cymene toward phenolic monoterpenoids (PM), namely, thymol and carvacrol.^37^ However, once EOs are extracted from the plant matrix, their chemical composition is no longer stabilized by cellular enzymatic control and becomes susceptible to extrinsic factors. Accordingly, the hypothesis proposed in this work is that exposure to heat and ultraviolet (UV) radiation provides sufficient energy to destabilize phenolic monoterpenoids, thereby promoting their dehydroxylation into lower-energy monoterpene hydrocarbons (as represented in Figure 3). This represents a reverse dynamic compared to the biosynthetic direction observed in plants, where the pathway proceeds from MH → PM.^37^ Postextraction, environmental stressors may drive the opposite transformations, PM → MH, particularly converting thymol and carvacrol back to their precursors *p*-cymene and γ-terpinene. This bidirectional interconversion highlights the potential to modulate the chemical composition of extracted essential oils through physical agents, with the aim of tailoring their bioactivity.

**Figure 3:**
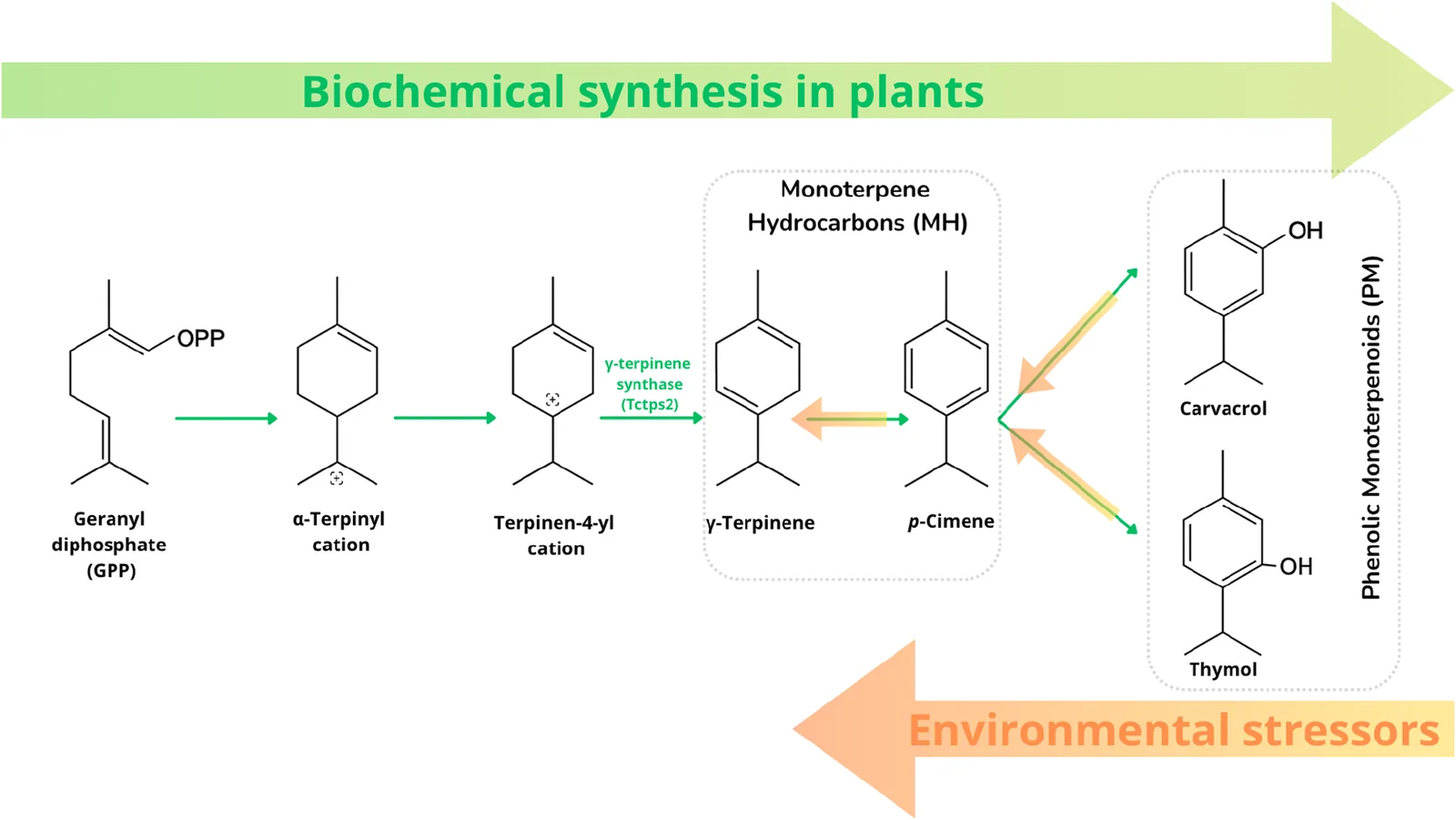
Proposed pathway under environmental stressors (temperature and UV radiation) in contrast to biosynthesis in plants. Green arrows represent *in situ* conversion steps, while orange arrows indicate stress-induced chemical processes in extracted essential oil components.

In the case of other alterations observed in different chemical groups, these may be associated with isomerization, polymerization (often evidenced by color changes), epoxidation, cyclization, oxidation, reduction, and cleavage reactions resulting from oxidative degradation or interactions among reactive functional groups. Such interactions may involve the presence of aldehydes/terpenoid aldehydes and alcohols/terpenoid alcohols, with the main changes occurring in reactive molecules such as unsaturated monoterpenes (e.g., myrcene, *p*-cymene, and γ-terpinene), aldehydes (α- and β-citral), highly reactive phenolics (thymol, carvacrol, and eugenol), and unsaturated sesquiterpenes such as caryophyllene (Table 2). However, no scientific references were found to support these implications for these essential oil compounds.

### 2.3 Effect of Photo-UV and Thermal Treatments on the Antibacterial Activity

The minimum inhibitory concentrations (MIC) of EOs subjected to control, photo-UV, and thermal treatments against *E. coli* and *S. aureus* are shown in Table 3 (Figure S5 shows the visual outcomes observed in the 96-well plates). The EOs from *O.* *vulgare* and *S. aromaticum* did not show changes in antibacterial activity under thermal or photoinduced degradation, maintaining similar MIC values across treatments. Additionally, *O.* *vulgare* exhibited the most consistent and effective inhibitory response against both bacteria among the tested oils.

**Table 3:** Minimum Inhibitory Concentration (MIC) of EOs against *E. coli* and *S. aureus* under Control, Photo-UV, and Thermal Treatments

		MIC [μL/mL]	
essential oils	treatments	*E. coli*	*S. aureus*
*Origanum vulgare* (OV)	control	1.50 > MIC > 0.75	1.50 > MIC > 0.75
	photo-UV	1.50 > MIC > 0.75	1.50 > MIC > 0.75
	thermal	1.50 > MIC > 0.75	1.50 > MIC > 0.75
*Cymbopogon citratus* (CC)	control	3.00 > MIC > 1.50	1.50 > MIC > 0.75
	photo-UV	(↓) **1.50 > MIC > 0.75**	1.50 > MIC > 0.75
	thermal	3.00 > MIC > 1.50	(**↑**) **3.00 > MIC > 1.50**
*Lippia sidoides* (LS)	control	3.00 > MIC > 1.50	3.00 > MIC > 1.50
	photo-UV	3.00 > MIC > 1.50	3.00 > MIC > 1.50
	thermal	3.00 > MIC > 1.50	(**↓**) **1.50 > MIC > 0.75**
*Syzygium aromaticum* (SA)	control	1.50 > MIC > 0.75	3.00 > MIC > 1.50
	photo-UV	1.50 > MIC > 0.75	3.00 > MIC > 1.50
	thermal	1.50 > MIC > 0.75	3.00 > MIC > 1.50

In contrast, the photoexposed essential oil of *C. citratus* showed increased antibacterial activity against *E. coli*, while its thermally treated form exhibited reduced effectiveness against *S. aureus*. Additionally, although the essential oil of *L. sidoides* showed increased activity against *S. aureus* after thermal treatment compared with the control and the photoexposed sample, it was overall the least effective essential oil against both bacterial species tested.

Although it is well-established that Gram-negative bacteria exhibit higher resistance to antimicrobial agents, primarily due to the lipopolysaccharide (LPS)-rich composition of their cell wall structure, this occurs because small hydrophilic molecules can diffuse through porin protein matrices that function as hydrophilic transmembrane channels.^42^ However, this differentiation in the response of Gram-negative and Gram-positive bacteria was not observed in the behavior of the essential oils tested. The essential oil of *O.* *vulgare* (the most effective) showed no variation even after exposure to physical agents, whereas the essential oil of *L. sidoides* (the least effective) exhibited similar responses against both bacterial species in its intact form and after photo-UV exposure. In contrast to this trend of effectiveness, *S. aromaticum* OE demonstrated greater effectiveness against the Gram-negative bacterium under all conditions evaluated.

Oregano EO rich in carvacrol (99.89%) demonstrated a MIC of 3.13 μL/mL against *S. aureus*.^43^ Similarly, oregano EO containing 57.71% carvacrol exhibited bactericidal activity against *S. aureus* at concentrations above 1.2 μL/mL, leading to a reduction of the cell population to <2 log CFU/mL after 120 min of exposure. Moreover, sub-inhibitory concentrations (0.03 and 0.015 μL/mL) of the essential oil were shown to suppress key physiological traits of this bacterium, such as coagulase and lipase production as well as salt tolerance, indicating a dose-dependent interference with microbial metabolism.^31^ For *E. coli*, MIC values of 219.9 and 236.1 mg/mL (corresponding to 0.233 and 0.250 μL/mL) were determined for oregano EO obtained from non-irrigated and irrigated plants, respectively.^42^

The *L. sidoides* essential oil, although it presented milder bactericidal activity among the EOs studied, exhibited a lower MIC than the 13 μL/mL reported by Castro et al.,^26^ for both bacteria. Veras et al.,^44^ studying *L. sidoides* with 84.9% thymol found the MIC for *S. aureus* of 128 μg/mL, indicating that the higher concentration of thymol increases the bactericidal effect, in comparison to the data of the present work.

The prevalence of the monoterpene aldehydes neral (α-citral) and geranial (β-citral), combined with the minority presence of myrcene (a monoterpene hydrocarbon), appears to enhance the antibacterial action of *C. citratus* essential oil. Similar to the findings in the present study, Lahyaoui et al.,^45^ identified 32.36% α-citral, 43.02% β-citral, and 4.3% myrcene, with a greater antibacterial effect against *S. aureus* (MIC of 0.2% v/v) than *E. coli* (MIC of 0.5% v/v). In contrast, Sherif et al.,^46^ identified pseudolimonene (19.2%), D-limonene (12.34%), α-terpinene (10.89%), α-terpinolene (5.53%), *m*-cymene (3.81%), and β-myrcene (2.87%) as the main constituents in *C. citratus* EO. As a consequence, a lower antimicrobial action was observed, with the highest concentration of EO analyzed (100 μL/mL) being unable to inhibit *S. aureus* completely.

The variations in the responses observed for *C. citratus* and *L. sidoides* may be explained by synergistic interactions (enhanced efficiency observed for CC-P and LS-T) or antagonistic interactions (reduced efficiency for CC-T) among major and minor compounds remaining after exposure. In *L. sidoides*, thymol, together with its derivatives *p*-cymene and γ-terpinene, contributes substantially to the overall bactericidal activity.^42^ However, the presence of umbellulone in the thermally treated EO may act as a synergistic agent, enhancing the bactericidal effect against *S. aureus*. Essential oils rich in this compound, such as the essential oil of *Poiretia bahiana*, exhibit significant inhibition against various bacteria, an effect associated with this constituent.^47^ For *C. citratus* EO, no specific compound alone accounts for the differences observed between the thermally and UV-treated oils. Nevertheless, small variations in the concentrations of verbenol, 4.8-dimethyl-3.7-nonadien-2-ol, geraniol, α-citral, and geranic acid may underlie the response variation observed against *E. coli* and *S. aureus*.

### 2.4 Antifungal Activity of Treated EOs

The analysis of antifungal activity revealed that, overall, *F. solani* exhibited greater resistance compared with *Colletotrichum* sp. (Figure 4). Among the EOs tested, fungal growth inhibition was most pronounced for oregano, followed by pepper-rosmarin, clove, and finally lemongrass. The high antifungal efficacy of oregano essential oil, the intermediate effect of clove essential oil, and the limited antifungal potential of lemongrass essential oil have already been reported.^27,28^ The positive effect of *L. sidoides* essential oil has also been previously demonstrated against *Colletotrichum* ^48^ and *Fusarium*.^49^

**Figure 4:**
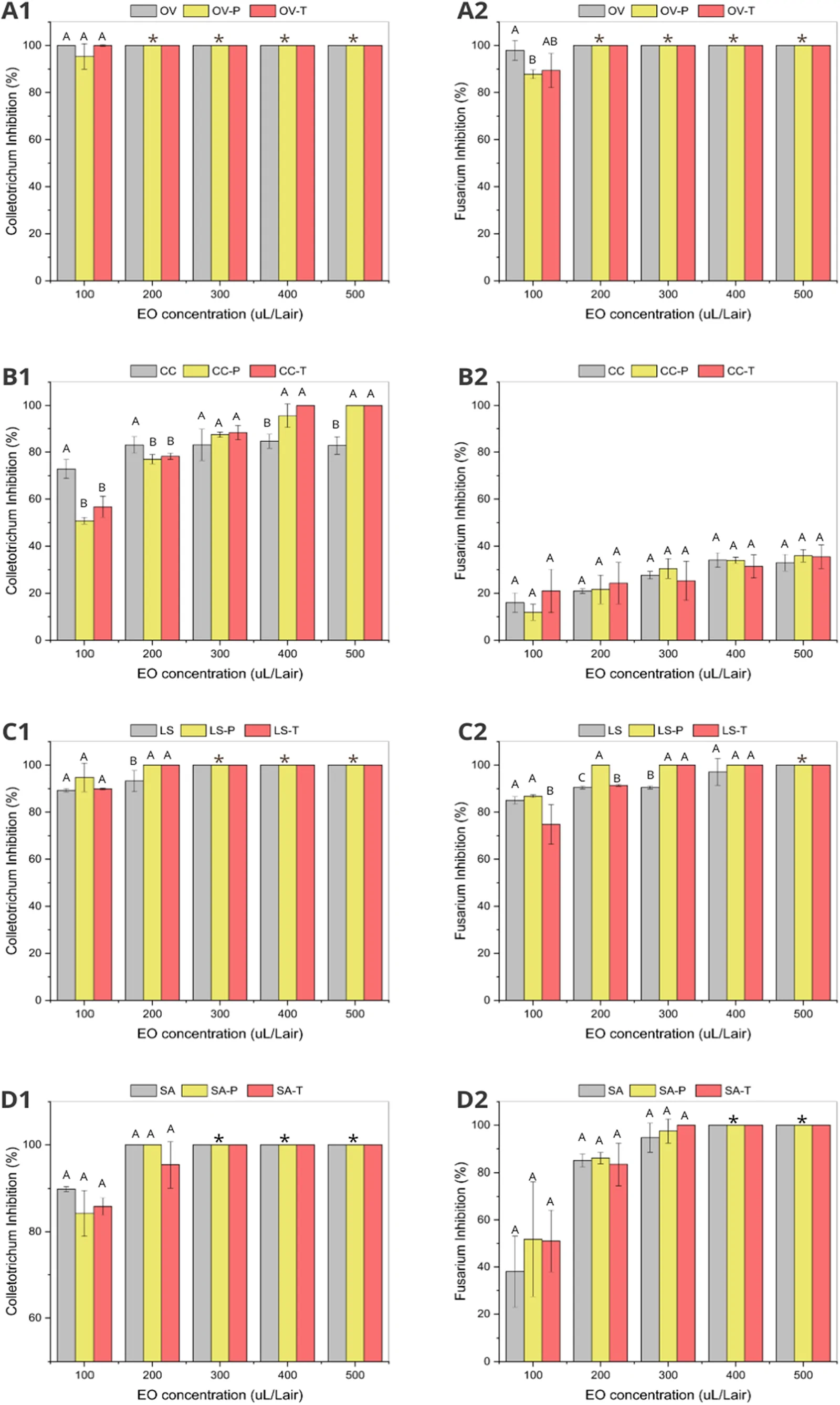
Percentage inhibition of non-, thermo-, and photo-UV treated essential oils, by volatile exposure, against *Colletotrichum* sp. and *Fusarium solani*. OV: *Origanum vulgare* intact (untreated); OV-P: *O. vulgare* photo-UV treated; OV-T: *O. vulgare* thermally treated. CC: *Cymbopogon citratus* intact (untreated); CC-P: *C. citratus* photo-UV treated; CC-T: *C. citratus* thermally treated; LS: *Lippia sidoides* intact (untreated); LS-P: *L. sidoides* photo-UV treated; LS-T: *L. sidoides* thermally treated; SA: *Syzygium aromaticum* intact (untreated); SA-P: *S. aromaticum* photo-UV treated; SA-T: *S. aromaticum* thermally treated. (*) Indicates complete inhibition of fungal growth at the evaluated essential oil concentration, regardless of the treatment. Different letters on the bars for each concentration indicate significant differences (*p* ≤ 0.05) according to ANOVA followed by Tukey’s test.

Considering the complete inhibition of *Colletotrichum* sp., significant differences in MIC were observed among untreated oils and those subjected to physical treatments. *O. vulgare* essential oil (OV) showed a MIC of 100 μL/L, whereas the UV-treated oil (OV-P) required 200 μL/L to achieve a similar effect. For *C. citratus* essential oil (CC), both UV-treated (CC-P) and heat-treated (CC-T) oils presented a MIC of 500 μL/L, while the untreated oil did not reach MIC within the concentrations tested. *L. sidoides* essential oil (LS) exhibited a MIC of 300 μL/L, while UV (LS-P) and thermal treatments (LS-T) reduced the MIC to 200 μL/L. In contrast, *S. aromaticum* essential oil (SA) presented a MIC of 200 μL/L, which increased to 300 μL/L after thermal treatment (SA-T). For *F. solani*, similar results were found for OV in all treatments; CC did not reach an inhibitory complete effect. The most relevant difference was observed for LS, while the untreated oil exhibited a MIC of 500 μL/L, LS-P and LS-T reduced the MIC to 200 and 300 μL/L, respectively. SA-T heat-treated showed a MIC of 300 μL/L superior to the others that reached 400 μL/L.

As previously discussed, the antimicrobial effects observed in EOs subjected to heat and photo-UV treatments may be related to changes in their chemical composition. For example, the decrease in monoterpene hydrocarbons and the increase in monoterpene aldehydes may be associated with the positive effect observed for thermal (CC-T) and photo-UV-treated (CC-P) lemongrass EO against *Colletotrichum* sp. Additionally, the reduction in sesquiterpene hydrocarbons observed in LS EO may be the reason for the decrease in the MIC found in the treated oils.

To further highlight the effects of the treatments on fungal morphology, scanning electron microscopy was performed at MIC values of LS, LS-T, and LS-P (Figure 5 *Coll* for *Colletotrichum* sp. and *Fusa* for *F. solani*). For both fungi, the images revealed marked morphological alterations among treatments. In the control group (untreated essential oil – 1D), hyphae displayed preserved morphology, with continuous surfaces and no apparent damage. The most pronounced morphological changes in *Colletotrichum* sp. were hyphal ruptures when exposed to untreated LS and LS-T (Figure 5 *Coll*, 2D and 4D), whereas for LS-P (Figure 5 *Coll* – 3D) the most notable alteration was hyphal shrinkage and twisting. In *F. solani*, LS and LS-P promoted spore (conidia) development and hyphal ruptures (Figure 5 *Fusa*, 2D and 3D), while LS-T induced more evident twisting, ruptures, and shrinkage.

**Figure 5:**
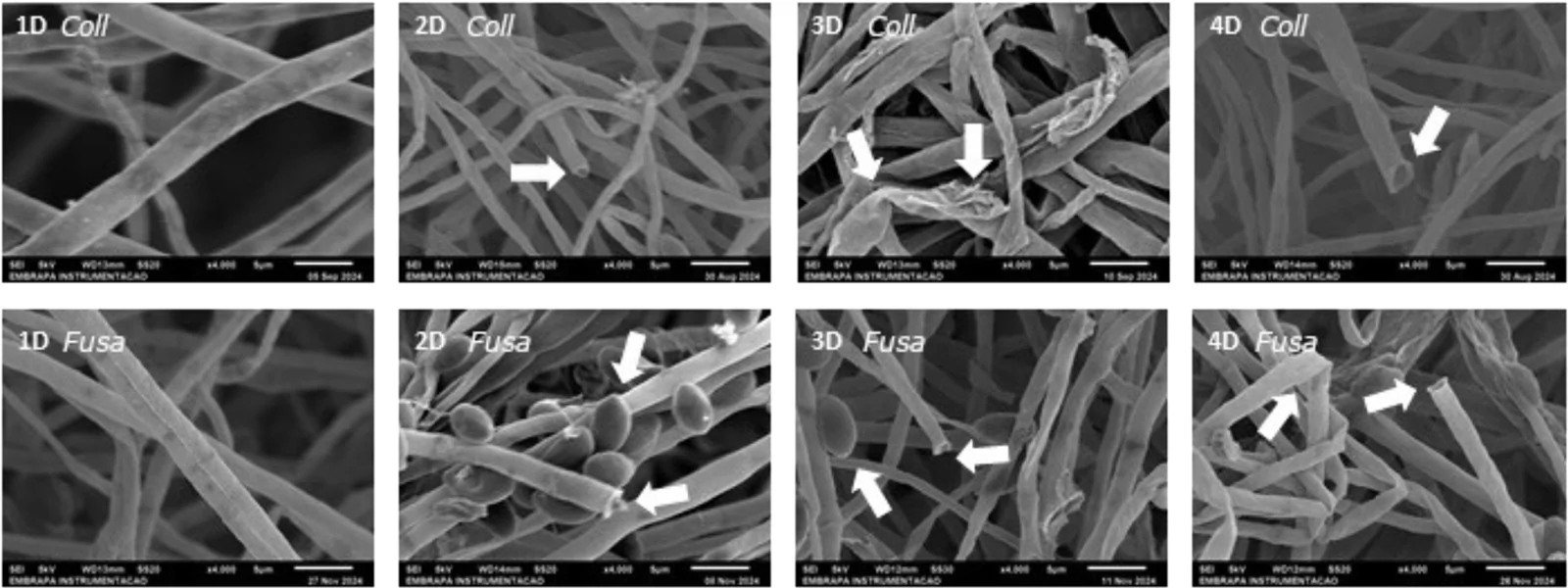
Scanning electron micrographs of *Colletotrichum* sp. (images with *Coll*) and *Fusarium solani* (images with *Fusa*) exposed to volatile compounds from *Lippia sidoides*. (1) Control; (2) untreated LS; (3) LS-P (photo-UV treated); and (4) LS-T (thermally treated). D indicates a magnification of 4000×. Arrows highlight the most evident morphological alterations observed among the treatments.

### 2.5 Effect of Treated EOs on Conidial Germination

Figure 6 shows the percentage of conidial germination under direct exposure to UV-treated (blue) and thermally treated (red) EOs compared with untreated EOs. For oregano essential oil against *Colletotrichum* sp. (A1-Figure 6), thermal treatment (OV-T) exhibited statistically higher inhibition of germination. However, considering the overall reduction trend, the dynamics across the three treatments were quite similar.

**Figure 6:**
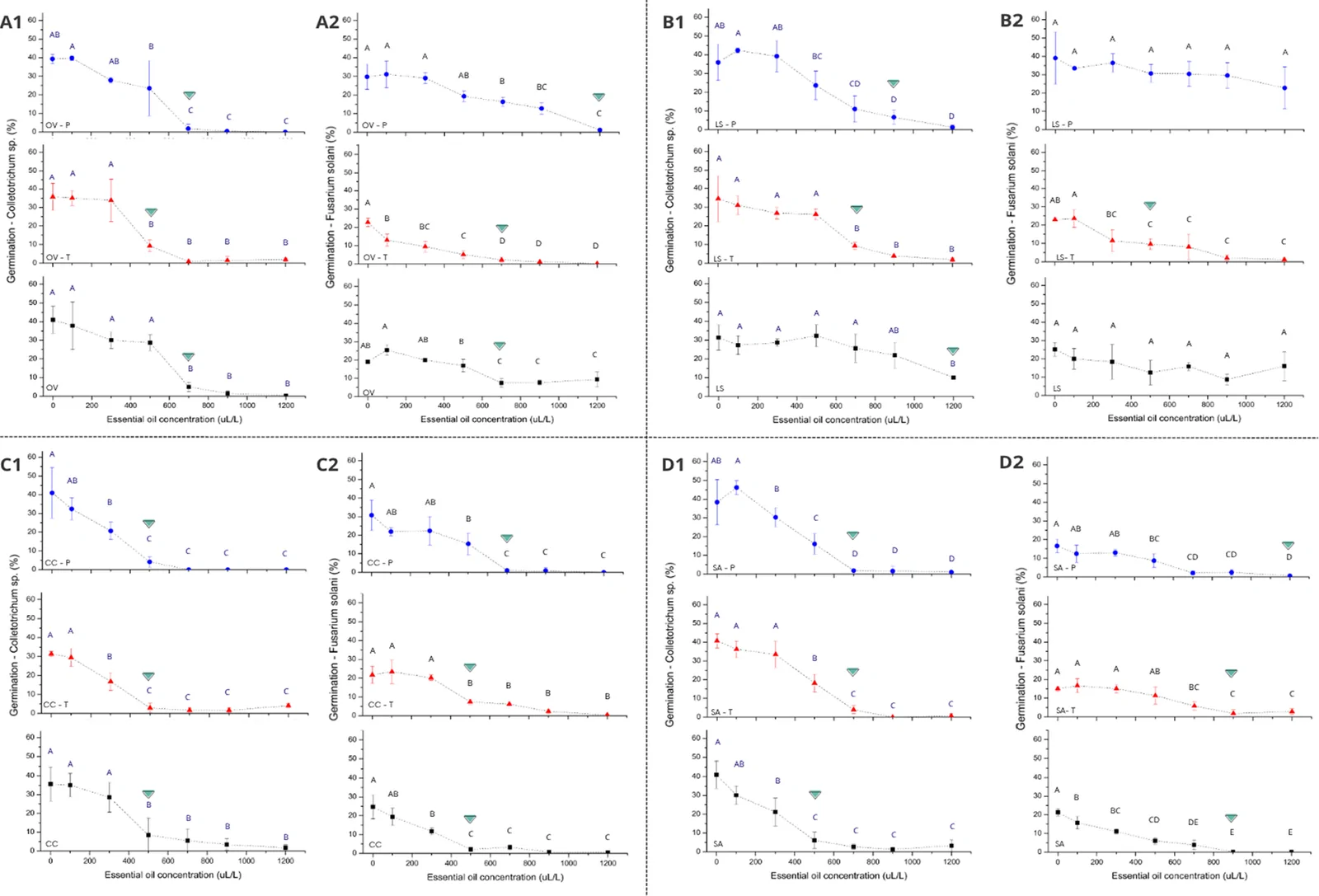
Percentage of conidial germination under direct exposure to untreated (black), UV-treated (blue) and thermally treated (red) essential oils. (A) *O. vulgare*; (B) *L. sidoides*; (C) *C. citratus*; (D) S. *aromaticum*. The graphic identification letters followed by the index [1] indicate antifungal potential against *Colletotrichu*m sp. and [2] against *Fusarium solani.* OV: *O. vulgare* intact (untreated); OV-P: *O. vulgare* photo-UV treated; OV-T: *O. vulgare* thermally treated. CC: *Cymbopogon citratus* intact (untreated); CC-P: *C. citratus* photo-UV treated; CC-T*: C. citratus* thermally treated; LS: *Lippia sidoides* intact (untreated); LS-P: *L. sidoides* photo-UV treated; LS-T: *L. sidoides* thermally treated; SA: *Syzygium aromaticum* intact (untreated); SA-P: *S. aromaticum* photo-UV treated; SA-T: *S. aromaticum* thermally treated. Different letters on the points for each concentration indicate significant differences (*p* ≤ 0.05) according to ANOVA followed by Tukey’s test. (▼) Highlights the concentration at which spore germination inhibition became most pronounced.

Against *F. solani* (A2- Figure 6), thermal treatment (OV-T) was the most effective, nearly abolishing germination at 700 μL/L, followed by UV treatment (OV-P) at 1200 μL/L, whereas the untreated EO did not achieve complete inhibition within the concentrations tested. For pepper-rosmarin EO against *Colletotrichum* (B1- Figure 6), thermal treatment (LS-T) was statistically superior. Nevertheless, both LS-T and LS-P achieved nearly complete inhibition at 1200 μL/L, in contrast to the untreated EO, which did not reach full inhibition at any concentration. When in contact with *F*. *solani* (B2- Figure 6), only LS-T induced marked reduction in germination, with values close to zero starting at 900 μL/L. For lemongrass essential oil against *Colletotrichum* (C1- Figure 6), the three treatments showed similar behavior, though CC-T and CC-P yielded lower germination values, approaching zero. In contrast, against *F. solani* (C2- Figure 6), physical treatments reduced essential oil efficacy: the untreated CC essential oil strongly inhibited germination at 500 μL/L, while CC-P and CC-T required higher concentrations (700 and 900 μL/L, respectively) to achieve similar inhibition. Finally, for clove essential oil against *Colletotrichum* (D1- Figure 6), all three treatments showed similar dynamics, with nearly complete inhibition at 700 μL/L. Against *F. solani* (D2- Figure 6), both untreated and thermally treated EOs displayed comparable dynamics, reaching near-complete inhibition at 900 μL/L, whereas UV treatment (SA-P) was more effective, achieving near-total inhibition at 700 μL/L.

These results indicate a sporostatic effect of the investigated essential oils, as treated conidia were not plated onto fresh culture media to determine whether the spore viability was irreversibly affected (sporocidal effect). Nevertheless, the findings provide further evidence that fungal responses to treated EOs may vary between spore germination and mycelial growth. This observation suggests that these essential oils can interfere with multiple stages of the fungal life cycle from the initiation of germination to hyphal development. It is also important to note that most previous studies have evaluated the effects of essential oils at a single concentration or have focused exclusively on germination inhibition. In contrast, studies monitoring both germ tube emergence and subsequent hyphae development across a range of concentrations remain scarce. Therefore, the concentration-dependent approach employed in the present study provides additional insights into the antifungal activity of non-treated and treated EOs during the early stages of fungal development.

Studies evaluating the effects of essential oils on the conidia of phytopathogenic fungi remain scarce. When reported, the inhibitory effects on conidial development are highly variable, similar to those observed for vegetative structures. Rozwalka et al.^50^ investigated the effects of the essential oils of *Cymbopogon martinii*, *Eugenia caryophyllata*, *Thymus vulgaris*, *Cinnamomum* sp., and *Cymbopogon citratus* on the microstructure of *C. gloeosporioides* and *C. musae* conidia. The authors observed that treatments with these essential oils induced distinct ultrastructural alterations, including varying degrees of vacuolization and cytoplasmic leakage. In addition, *S. aromaticum* EO, rich in eugenol, completely inhibited the germination of *F. solani* spores at 250 μL/L, whereas the commercial fungicide Ridomil achieved total inhibition only at 1600 μL/L. These findings highlight the potential of essential oils as promising alternatives to conventional fungicides.^51^ However, reports evaluating the effects of thermally treated or UV-exposed essential oils on fungal spore germination and development are currently lacking.

The variations observed in the present study, associated with both the essential oil treatment and the fungal species evaluated, may be related to differences in the solubility and bioavailability of essential oil constituents as well as their interactions with the cytoplasmic membrane, which ultimately determine antifungal efficacy. Furthermore, the thickness and composition of the spore wall may influence the susceptibility of the fungal propagules to antifungal compounds. These findings confirm that fungal susceptibility to EOs is species dependent. Moreover, they demonstrate that physical treatments applied to EOs may either enhance or diminish antifungal activity depending on the oil type and the target microorganism. In the specific case of *L. sidoides* essential oil, the results confirm that physical treatments not only modulate its bioactivity but also alter its mode of action on fungal structures.

## 3 Conclusions

This study demonstrated that postextraction physical treatments, specifically heating and UV exposure, can effectively modulate the chemical composition and antimicrobial activity of essential oils. Thermal and photochemical stresses led to distinct alterations in chemical profiles, particularly in *L. sidoides* and *O. vulgare* essential oils, where reductions in phenolic compounds were accompanied by an increase in hydrocarbon terpenes. Among the evaluated oils, *S. aromaticum* exhibited the greatest chemical stability, although there was a pronounced color variation after thermal treatment. These compositional shifts were closely associated with improved antibacterial and antifungal activities, notably in UV-treated *C. citratus* and thermally treated *L. sidoides* essential oils, which exhibited enhanced efficacy against both Gram-positive and Gram-negative bacteria. Furthermore, thermally and UV-treated essential oils also exhibited enhanced activity against mycelial growth and conidial germination of *Colletotrichum* sp. and *F. solani*.

Collectively, these findings highlight that controlled postextraction physical treatments constitute a simple yet powerful strategy to enhance the bioactivity of essential oils. Moreover, the observed effects reinforce the biotechnological potential of Brazilian native *L. sidoides* as an underexplored antimicrobial source. The capacity to enhance the antimicrobial properties through adjustments in the temperature and UV exposure can offer a practical approach. As the current literature provides very limited evidence on postextraction physical modification of essential oils, this study establishes a conceptual and experimental foundation for future research aimed at optimizing physicochemical parameters to develop naturally derived, broad-spectrum antimicrobial agents through physical modulation of essential oils.

## 4 Material and Methods

### 4.1 Materials and Microbial Resources

The EOs of oregano (*O. vulgare* L.) from France and clove (*S. aromaticum*) from Madagascar were obtained from Harmonie Aromaterapia (Florianópolis, SC, Brazil). Lemongrass (*C. citratus*) essential oil, sourced from Brazil, was purchased from Laszlo Aromas e Sabores (Belo Horizonte, MG, Brazil), while pepper-rosmarin (*L. sidoides*) essential oil [SISGEN No. ACDAA33], also from Brazil, was kindly donated by Legeé Aromas (Estiva Gerbi, SP, Brazil). Freshly produced essential oils were sourced to minimize pre-experimental degradation. Upon arrival, OEs from the same batch were immediately exposed to thermal and UV treatments for 48 h. Both treated samples and untreated controls were analyzed immediately following treatment to ensure direct batch composition comparisons. All samples were subsequently refrigerated at 5 °C to preserve chemical integrity for subsequent analyses.

The effects of the EOs were tested against the bacteria *E. coli* ATCC 25922 and *S. aureus* subsp. *aureus* ATCC 25923. Additionally, the fungi *F. solani* IMI 314228 CCT 2876, donated by the Strains Bank of the Tropical Culture Collection (CCT) at the André Tosello Foundation for Research and Technology (Campinas, São Paulo, Brazil), and *Colletotrichum* sp. (isolated from *Olea europaea* L. in Maria da Fé, Minas Gerais, Brazil) [SISGEN No. ACDAA33], donated by the Microorganism Collection of Embrapa Pecuária Sudeste—CMEPS (São Carlos, São Paulo, Brazil), were also included in the study.

The culture media Potato Dextrose Agar (PDA) K25-1022, Brain Heart Infusion Broth (BHI) K25-1400, Bacteriological Peptone K25-611701, and European Bacterial Agar K25-1800 were obtained from KASVI (Madrid, Spain). Tween 80 was purchased from Synth (São Paulo, Brazil); Dextrose monohydrate PA 14431-43-7 from Êxodo Científica (São Paulo, Brazil); sodium chloride 31434, 2,3,5-triphenyl tetrazolium chloride (TTC) T8877 and Streptomycin Sulfate S6501 from Sigma-Aldrich (Missouri). The other standard reagents were of analytical grade or superior.

### 4.2 Essential Oil Exposure to Thermal and Photo-UV Conditions

The accelerated chemical modulation of EOs from *O. vulgare* (oregano), *C. citratus* (lemongrass), *S. aromaticum* (clove), and *L. sidoides* (pepper-rosmarin) was conducted through photo-UV and thermal exposure over 48 h. Following the method described by Procopio et al.,^4^ 2.0 g of each essential oil was placed in transparent 20 mL chromatography vials, sealed with crimp caps, and silicone septa. For thermal alterations, duplicates of each essential oil were incubated in a drying oven (Nova Ética, Brazil) without air circulation at 70 °C. Additionally, photo-UV alterations were induced by incubating the duplicate vials in a photosensitization chamber equipped with 4 UV lamps (Philips 15 W G15T8) at a controlled temperature of 15 °C. After exposure, all samples were stored in dark conditions at ambient temperature until chemical characterization and microbiological analysis.

### 4.3 Essential Oil Color Variation after Thermal and Photo-UV Exposure

For photographic documentation and color change quantification, approximately 1.0 mL of the EOs of *O. vulgare*, *C. citratus*, *S. aromaticum*, and *L. sidoides* were transferred into 2.0 mL, 12 × 32 mm clear glass vials (Varian—Screw Top Standard Opening Vial Kit) after 48 h of thermal (T) and photo-UV (P) stress exposure. These samples were evaluated using a Konica Minolta CR-400 colorimeter (Konica Minolta, Osaka, Japan), equipped with a C 2° illuminant and employing the CIELAB color space (L*: brightness, a*: green (−) to red (+), and b*: blue (−) to yellow (+)).^4^ For color measurement, each vial was placed directly at the colorimeter’s reading port without a cap to prevent color interference. Each sample was analyzed in quadruplicate. The total color difference (Δ*E*) was calculated relative to the non-exposed essential oil, using eq 1, as follows:

1 $$\Delta E^{*}=\sqrt{{\left(L_{ex}^{*}-L_{0}^{*}\right)}^{2}+{\left(a_{ex}^{*}-a_{0}^{*}\right)}^{2}+{\left(b_{ex}^{*}-b_{0}^{*}\right)}^{2}}$$

where *L*_0_*, *a*_0_*, and *b*_0_* correspond to the values of the unexposed essential oil, and *L*_ex_*, *a*_ex_*, and *b*_ex_* refer to the parameters measured after 48 h of exposure to each physical treatment.

### 4.4 Essential Oil Chemical Characterization by Gas Chromatography Coupled to Mass Spectrometry

The EO composition was accessed by a Shimadzu GC-2010 Plus system (Kyoto, Japan) coupled with a quadrupole mass spectrometer (GC-MS), based on the methodology described by Fukuyama et al.^27^ A non-polar DB-5MS capillary column (30 m × 0.25 mm, i.d. × 0.25 μm) was employed, with helium as the carrier gas at a 1 mL/min flow rate. Samples were diluted in dichloromethane (10% v/v) and injected (0.5 μL) in split mode (1:100). The injector temperature was set to 250 °C, and the oven temperature was programmed from 60 to 240 °C at a rate of 3 °C/min; the interface temperature was 240 °C, and the ion source operated at +70 eV, with an m/z range of 35–350. The linear temperature-programmed retention index (RI) was calculated using an alkane solution (C8–C20, Sigma-Aldrich). The analytes were identified by comparing their retention indices (RI) and mass spectra with reference data from the literature. A minimum similarity of 85% in the mass spectra and a maximum RI deviation of ±10 were considered acceptable for identification. When available, authentic standards were co-injected to confirm the compound identities. For the semiquantitative analysis of the EOs based on relative peak areas, the same gas chromatography system was employed, this time coupled to a flame ionization detector (GC-FID). All qualitative and semiquantitative determinations were carried out in triplicate.

### 4.5 Minimum Inhibitory Concentration (MIC) against Bacteria

The antimicrobial activity of the intact, UV-exposed, and thermally treated EOs against *E. coli* and *S. aureus* was evaluated using the micro-scale minimum inhibitory concentration (MIC) assay, following the CLSI M07 guideline for dilution antimicrobial susceptibility testing of aerobic bacteria, with minor modifications. A bacterial cell suspension was prepared with 5 mL of BHI broth inoculated with a loopful of colonies grown on BHI agar and incubated at 37 °C overnight. Using the McFarland standard 0.5 at 625 nm, the inocula of both bacteria were adjusted to 10^6^ cells/mL.

Ninety-six well microplates were prepared with 100 μL of BHI broth with Tween 80 (0.5%) in all wells, except for wells 4, 8, and 12 of row A, and the entire row D (Figure 7). Negative controls were applied in wells 1–3 of row A without bacterial inoculation. Positive controls were performed in wells 5–7 of row A inoculated with 10 μL of *E. coli*, and wells 9–11 inoculated with 10 μL of *S. aureus*. Bactericidal controls were prepared with serial dilutions of streptomycin (initial concentration 20.00 mg/mL) in rows B and C, followed by the addition of 10 μL of *E. coli* to row B and 10 μL of *S. aureus* to row C. Row D remained empty. Control of EOs microbial contamination was prepared in row E with a serial dilution of 100 μL of EOs (initial concentration 6.00 μL/mL) into wells 1–6 and 7–12, without bacterial suspension. The effective bacterial sensitization with EOs was carried out in rows F, G, and H with the same process as row E, followed by adding 10 μL of bacterial inoculum of *E. coli* to wells 1–6 and *S. aureus* to wells 7–12. Considering the dilutions, concentrations of 3.0000, 1.5000, 0.7500, 0.3750, 0.1875, and 0.09375 μL/mL of each essential oil were tested against both bacteria.

**Figure 7:**
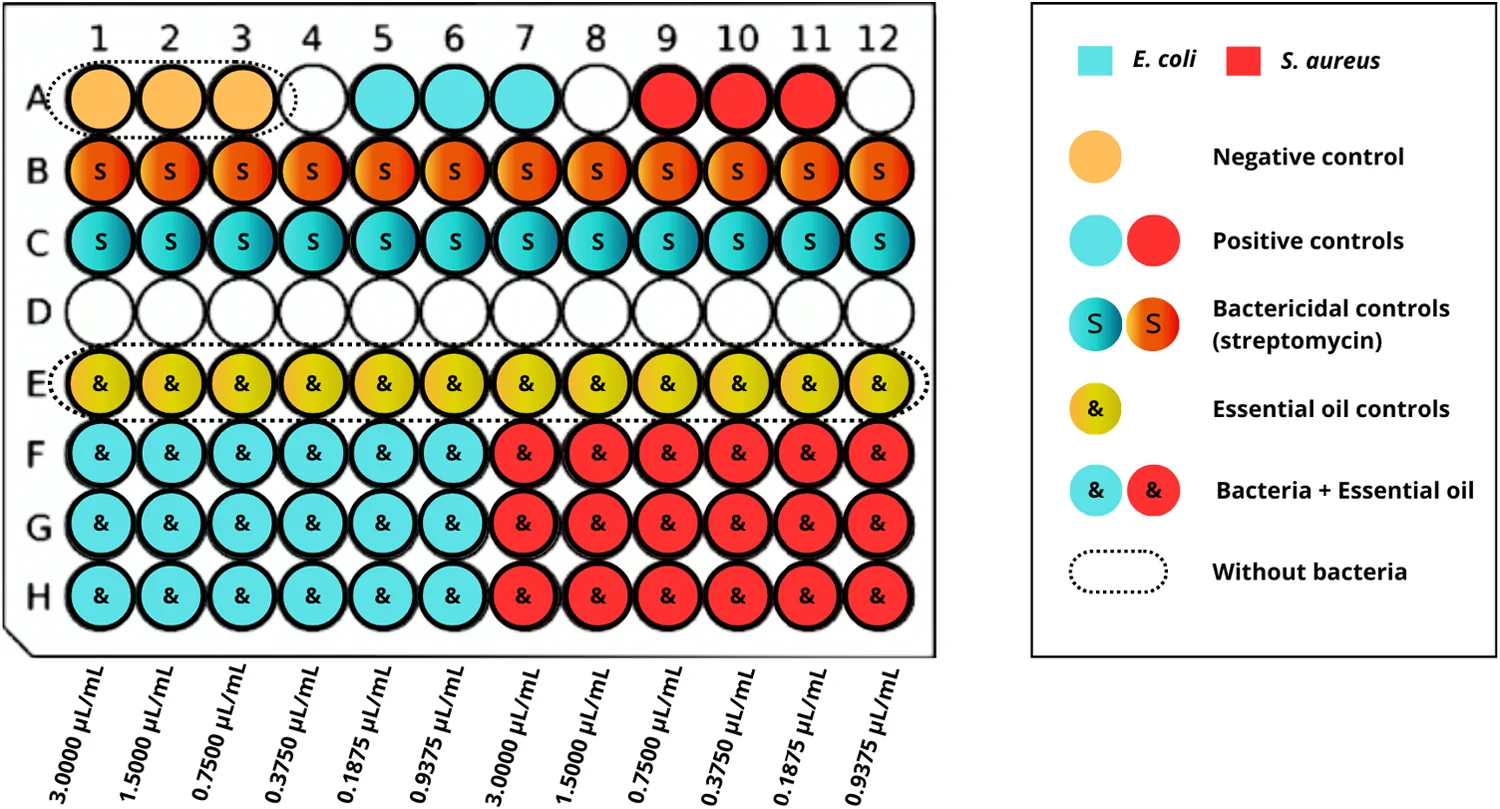
Schematic representation of the micro-scale minimum inhibitory concentration (MIC) method for bacteria.

After preparation, the microplates were incubated at 37 °C for 24 h. Subsequently, 40 μL of a 0.1% 2,3,5-triphenyl tetrazolium chloride solution was added to all wells, and the plates were incubated for an additional hour at 37 °C. A red-pink coloration identified bacterial growth.

### 4.6 Antifungal Activity by EO Volatile Exposure

The antifungal activity of the intact, UV-exposed, and thermally treated EOs was tested using the volatile exposure method according to Fukuyama et al.^27^ Concentrations of 100, 200, 300, 400, and 500 μL of EOs per liter of air were used against *Colletotrichum* sp. and *F. solani*.

Petri dishes (8 cm in diameter) were prepared with 20 mL of PDA medium, ensuring a headspace volume of approximately 50 mL. Sterile filter paper disks (2 cm in diameter) were affixed to the center of each Petri dish lid. Fungal inoculation was performed using a 9 mm mycelial plug of each fungus, and the EOs was applied to the filter paper disc. The dishes were sealed with parafilm and incubated at 28 °C. After 7 days of incubation, the colony growth was marked and measured with a caliper Digital ABSOLUTE AOS Serie 500 Digimatic (Mitutoyo, Japan). The Petri dish was divided into meridians spaced 45° apart (resulting in four sections), and the colony diameter growth was assessed by averaging the measurements taken from each section of the dish. All assays were conducted in quadruplicate, and a control group without EOs application was included. The minimum inhibitory concentration (MIC) of EO volatiles was defined as the lowest concentration at which all four Petri dishes showed 100% fungal growth inhibition.

#### 4.6.1 Scanning Electron Microscopy (SEM)

To investigate morphological alterations at the microscopic level resulting from exposure to EOs volatiles, the essential oil that exhibited the greatest visual, chemical, and antifungal changes compared to the untreated essential oil was selected for SEM imaging of *Colletotrichum* sp. and *F. solani*. Potato dextrose agar (PDA) medium was inoculated with an agar plug containing fungal mycelium and incubated at 28 °C for 4 days. For the volatile exposure, intact and previously exposed EOs, at MIC concentration, were individually applied to filter paper disks affixed to the Petri dish lids. The exposure method was carried out in triplicate, alongside a control group without essential oil treatment. For the stub preparation, 5 mm mycelial disks were excised from the cultures and fixed overnight in 3% (v/v) glutaraldehyde after 48 h of incubation. Subsequently, the samples were rinsed in a phosphate buffer (0.05 M, pH 6.8) and progressively dehydrated through an acetone gradient (30, 50, 70, 90, and 100% v/v). After dehydration, the samples underwent critical point drying using liquid CO_2_. Finally, the dried disks were affixed to SEM stubs with double-sided adhesive tape, coated with a thin layer of gold, and examined using a scanning electron microscope (JEOL JSM-6701F, Tokyo, Japan).

### 4.7 Conidial Germination Assay

In addition to evaluating the effects of treated EOs on fungal structures developed as mycelia, this study also investigated their impact on the germination of conidia, which are fungal resistance structures. Conidial suspensions were prepared following the methodology described by Mello et al.,^52^ with slight modifications. *Colletotrichum* sp. and *F. solani* strains were subcultured on Potato Dextrose Agar (PDA) plates supplemented with streptomycin (1 mg/mL) and incubated at 30 °C for 10 to 20 days, until conidial formation was confirmed by light microscopy. After conidial development, 3.0 mL of sterile saline solution (0.9% NaCl) containing 0.5% Tween 80 was added to each plate. The mycelium with conidia was then gently scraped using a Drigalski spatula. The resulting suspension was transferred to a 15 mL Falcon tube and supplemented with streptomycin (1 mg/mL). Conidial concentration was determined using a Neubauer chamber and adjusted to 10^7^ conidia/mL, then stored in a 50 mL Falcon tube for subsequent use.

Germination assays were carried out based on the method proposed by Pimentel et al.,^53^ with adaptations. A 10 μL aliquot of conidial suspension (10^8^ conidia/mL) was incubated in 2000 μL of Sabouraud medium supplemented with 0.05% (v/v) Tween 80 and EOs solutions at different concentrations (100, 300, 500, 700, 900, and 1200 μL/L). Sabouraud medium with 0.05% Tween 80 (without essential oil) served as the positive control. Samples were incubated at 28 °C for 24 h, and germination inhibition was assessed using light microscopy (Optical Systems Nova 107). All germination assays were performed in triplicate.

### 4.8 Statistical Analysis

Statistical analysis was performed using the trial version of Minitab software (version 16.1.0, Minitab Inc., State College, PA). Analysis of variance (ANOVA) followed by Tukey’s post hoc test was conducted at a 5% significance level (*p* < 0.05).

## Supplementary Material



## Notes

Data are provided regarding representative chromatograms of oregano (*O. vulgare*) essential oil (OEOV), lemongrass (*C. citratus*) essential oil (OECC), pepper-rosmarin (*L. sidoides*) essential oil (OELS), and clove (*S. aromaticum*) essential oil (OESA) under control conditions, thermal postextraction treatment (70 °C, 48 h), and UV irradiation postextraction treatment (48 h), including the major compounds identified in each essential oil, as well as visual appearances of 96-well microplate assays showing the antibacterial activity of the tested essential oils against *Staphylococcus aureus* and *Escherichia coli* (PDF)
