# Supplementary material for: Can Temperature and UV Exposure Alter the Antimicrobial Properties of the Essential Oils? An Investigation of Origanum vulgare, Cymbopogon citratus, Syzygium aromaticum, and Lippia sidoides
Source: ACS Omega. 2026 Sep 16;11(38):57727–41. doi: 10.1021/acsomega.6c06182 (PMC13629405; doi:10.1021/acsomega.6c06182)
Supplement: Supplementary file 1 [file ao6c06182_si_001.pdf]

CAN TEMPERATURE AND UV EXPOSURE ALTER THE ANTIMICROBIAL PROPERTIES OF THE ESSENTIAL OILS? AN INVESTIGATION OF *ORIGANUM VULGARE*, *CYMBOPOGON CITRATUS*, *SYZYGIUM AROMATICUM,* AND *LIPPIA SIDOIDES*

Ramon Peres Brexó[1], Conny Wara Teran Fukuyama[2], Giulia Suguimoto Martins[2], Maria Eduarda da Mata Martins[3], Fernanda Ramalho Procopio[1], Stanislau Bogusz Junior[4], Marcos David Ferreira[1*]

1- Brazilian Agricultural Research Corporation, Embrapa Instrumentation, São Carlos 13561-206, Brazil.
2- Center for Biological and Health Sciences, Federal University of São Carlos (UFSCar), São Carlos, SP, Brazil.
3- Postgraduate Program in Food Science and Engineering, IBILCE, São Paulo State University "Julio de Mesquita Filho", UNESP, São José do Rio Preto, SP, 15054-000, Brazil.
4- University of São Paulo (USP), São Carlos Institute of Chemistry (IQSC), São Carlos, São Paulo 13566-590, Brazil.

Supporting Information

- Number of page: 6
- Number of figures: 5

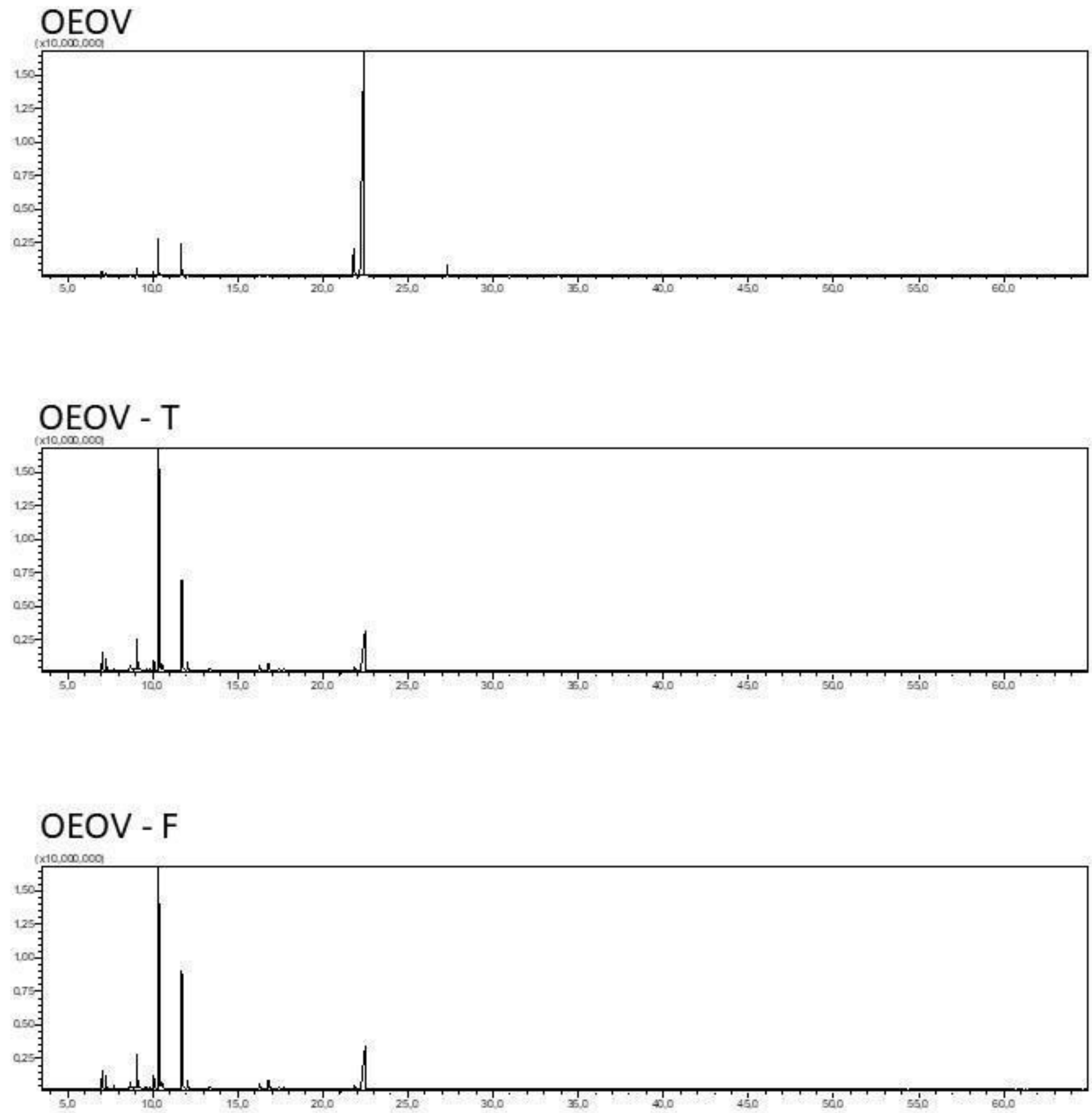

Figure S1. Representative chromatograms of oregano (*O. vulgare*) essential oil (OEOV) control, under thermal post-extraction treatment - 70 °C, 48 h (OEOV -T), and UV irradiation post-extraction treatment - 48 h (OEOV -F).

OEOV: carvacrol (79.93%)

OEOV - T: p-cymene (39.43%), carvacrol (20.84%), and γ-terpinene (18.15%)

OEOV - F: p-cymene (37.29%), carvacrol (20.35%), and γ-terpinene (19.68%)

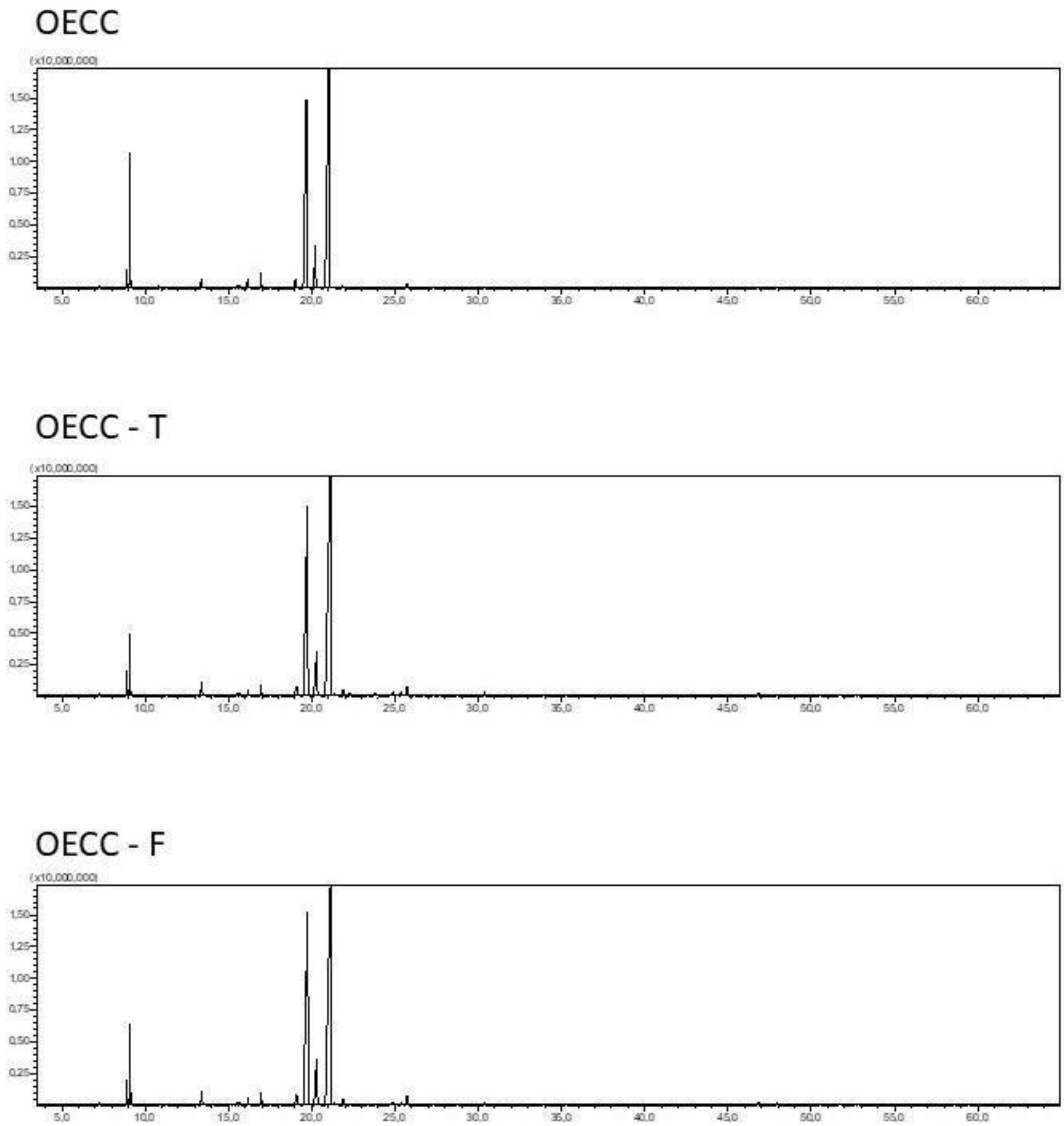

Figure S2. Representative chromatograms of lemongrass (*C. citratus*) essential oil (OECC) control, under thermal post-extraction treatment - 70 °C, 48 h (OECC -T), and UV irradiation post-extraction treatment - 48 h (OECC -F).

OECC: β-citral (42.46%), α-citral (33.27%), and myrcene (10.03%)

OECC - T: β-citral (45.71%), α-citral (34.22%), and myrcene (3.87%)

OECC - F: β-citral (44.75%), α-citral (34.39%), and myrcene (3.95%).

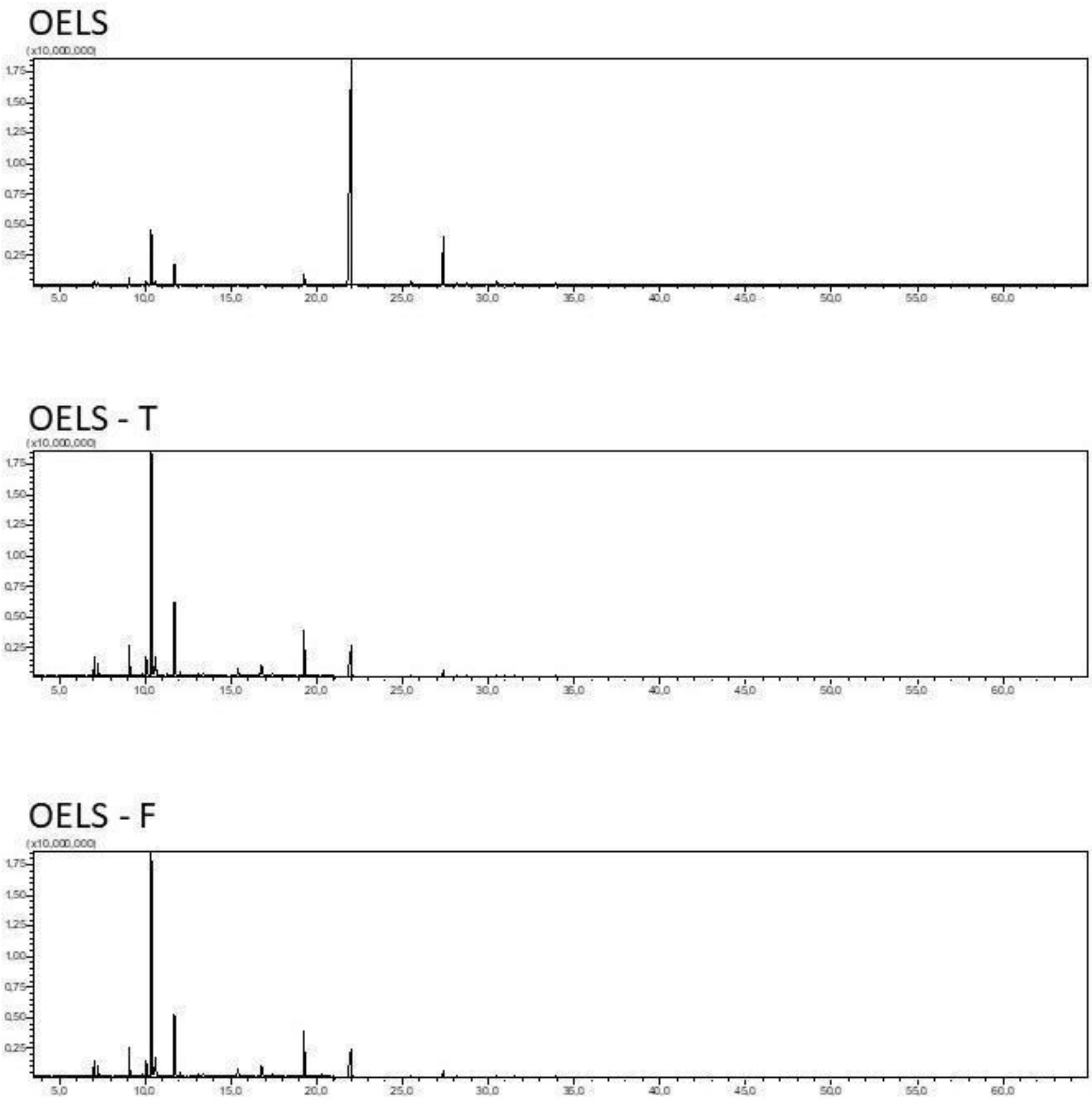

Figure S3. Representative chromatograms of pepper-rosmarin (*L. sidoides*) essential oil (OELS) control, under thermal post-extraction treatment - 70 °C, 48 h (OELS -T), and UV irradiation post-extraction treatment - 48 h (OELS -F).

OELS: thymol (61.90%), caryophyllene (10.56%), and p-cymene (9.27%)

OELS - T: p-cymene (38.93%), γ-terpinene (13.25%), and thymol (11.76%)

OELS - F: p-cymene (40.81%), carvacrol (11.94%), and thymol (11.84%).

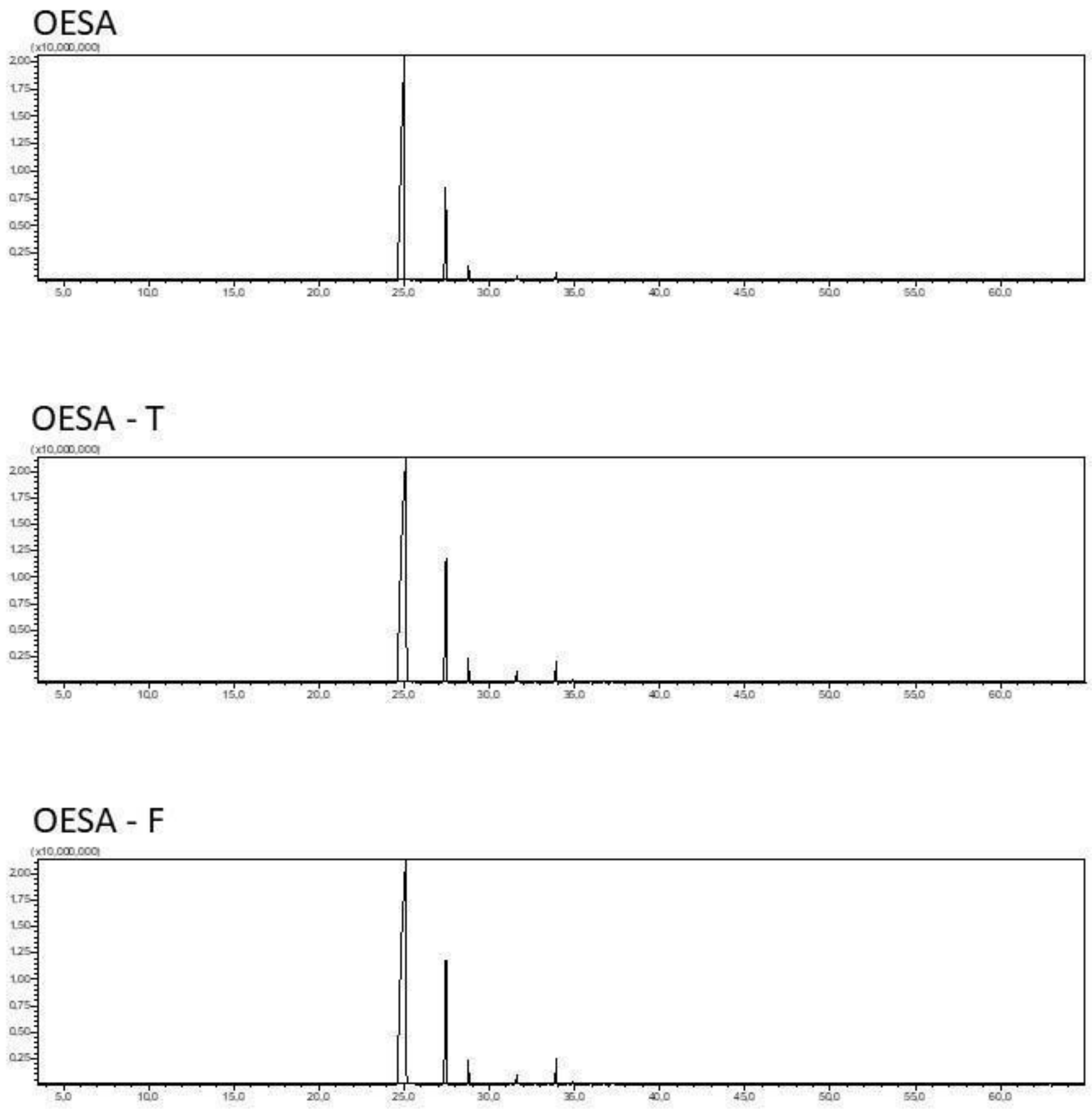

Figure S4. Representative chromatograms of clove (*S. aromaticum*) essential oil (OESA) control, under thermal post-extraction treatment - 70 °C, 48 h (OESA -T), and UV irradiation post-extraction treatment - 48 h (OESA -F).

OESA: eugenol (84.50%), and caryophyllene (11.68%)

OESA - T: eugenol (82.54%), and caryophyllene (12.03%)

OESA - F: eugenol (82.46%),and caryophyllene (11.62%).

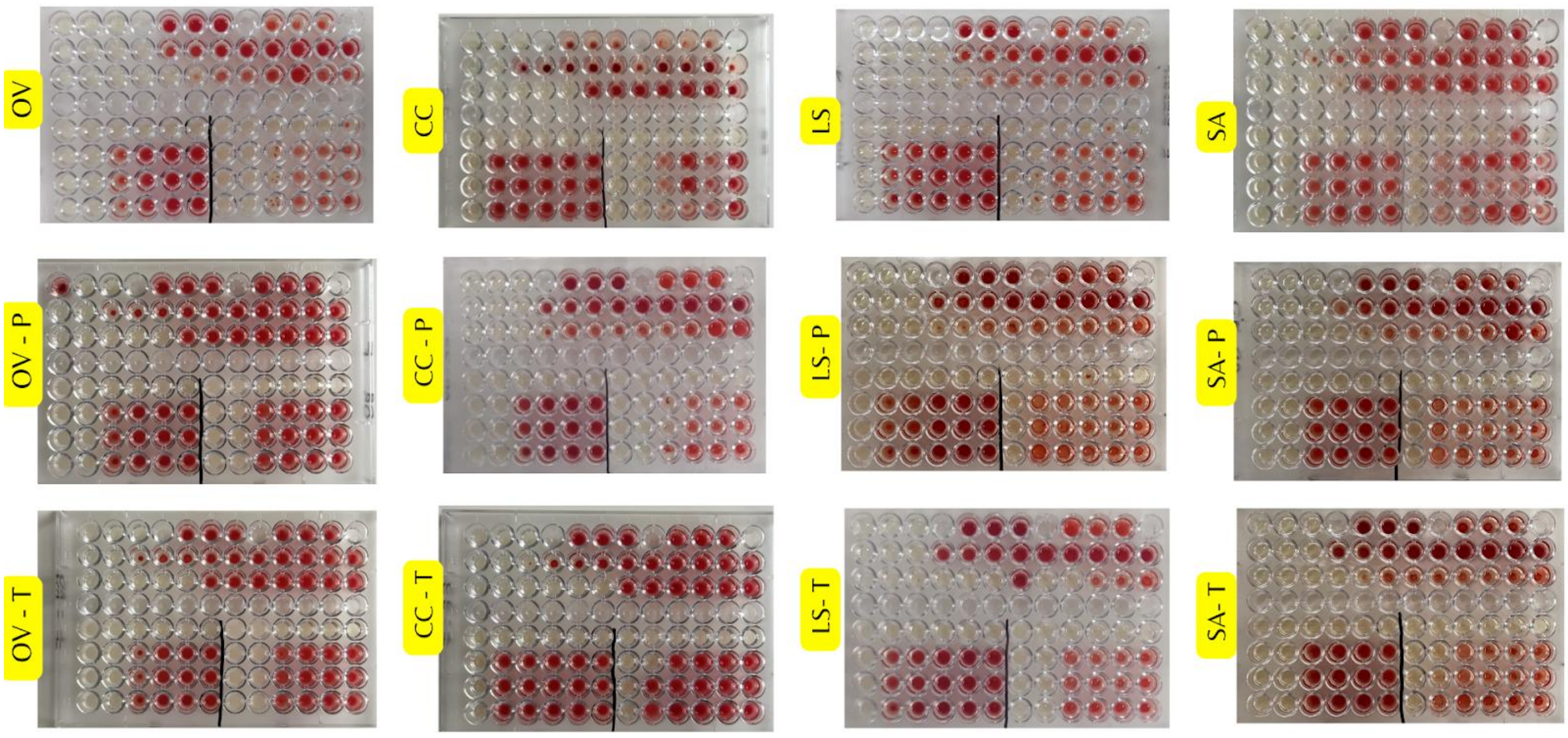

Figure S5. Visual appearance of 96-well microplate assays showing the antibacterial activity of the tested essential oils against *Staphylococcus aureus* and *Escherichia coli*. OV – oregano; CC – lemongrass; LS – pepper-rosmarin; SA – clove. P – photo-UV treated; T – thermally treated. Red-colored wells denote bacterial growth, while translucent wells indicate total inhibition of bacterial metabolic activity.
